# A New Force Field for OH^–^ for Computing Thermodynamic and Transport Properties of H_2_ and O_2_ in Aqueous NaOH and KOH Solutions

**DOI:** 10.1021/acs.jpcb.2c06381

**Published:** 2022-11-03

**Authors:** Parsa Habibi, Ahmadreza Rahbari, Samuel Blazquez, Carlos Vega, Poulumi Dey, Thijs J. H. Vlugt, Othonas A. Moultos

**Affiliations:** † Engineering Thermodynamics, Process & Energy Department, Faculty of Mechanical, Maritime and Materials Engineering, Delft University of Technology, Leeghwaterstraat 39, 2628 CBDelft, The Netherlands; ‡ Department of Materials Science and Engineering, Faculty of Mechanical, Maritime and Materials Engineering, Delft University of Technology, Mekelweg 2, 2628 CDDelft, The Netherlands; § Depto. Química Física, Fac. Ciencias Químicas, Universidad Complutense de Madrid, 28040Madrid, Spain

## Abstract

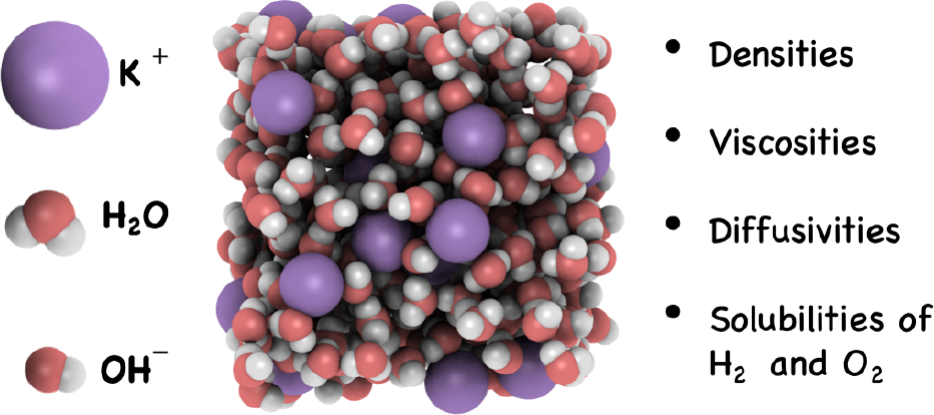

The thermophysical properties of aqueous electrolyte solutions are of interest for applications such as water electrolyzers and fuel cells. Molecular dynamics (MD) and continuous fractional component Monte Carlo (CFCMC) simulations are used to calculate densities, transport properties (i.e., self-diffusivities and dynamic viscosities), and solubilities of H_2_ and O_2_ in aqueous sodium and potassium hydroxide (NaOH and KOH) solutions for a wide electrolyte concentration range (0–8 mol/kg). Simulations are carried out for a temperature and pressure range of 298–353 K and 1–100 bar, respectively. The TIP4P/2005 water model is used in combination with a newly parametrized OH^–^ force field for NaOH and KOH. The computed dynamic viscosities at 298 K for NaOH and KOH solutions are within 5% from the reported experimental data up to an electrolyte concentration of 6 mol/kg. For most of the thermodynamic conditions (especially at high concentrations, pressures, and temperatures) experimental data are largely lacking. We present an extensive collection of new data and engineering equations for H_2_ and O_2_ self-diffusivities and solubilities in NaOH and KOH solutions, which can be used for process design and optimization of efficient alkaline electrolyzers and fuel cells.

## 1 Introduction

Modeling aqueous alkaline solutions is of interest for a broad array of manufacturing and separation processes.^1,2^ Aqueous alkaline solutions containing potassium hydroxide (KOH) and sodium hydroxide (NaOH) are often used for electrolysis and in fuel cells due to their high ionic conductivities and low cost.^3−7^ NaOH and KOH have solubilities exceeding 18 mol/kg in water at 293 K (and above)^8,9^ and significantly influence the thermophysical properties of the solution.^10^ The interplay between different thermophysical properties (e.g., densities, viscosities, and ionic conductivities) of aqueous NaOH and KOH solutions influences the product gas purity, and the energy (and Faradaic) efficiency of alkaline-water electrolyzers.^11−13^ Knowledge of the thermodynamic and transport properties of hydrogen (H_2_) and oxygen (O_2_) gas in aqueous NaOH and KOH solutions is therefore highly relevant for optimization and process design of electrolyzers.^12,14^

Modeling electrolyte systems is a challenging endeavor because of the strong long-range ionic interactions, which make the solutions highly nonideal.^1,15−17^ Electrolyte solutions are commonly modeled using semiempirical equations of state and molecular based simulations.^1,15−26^ Semiempirical equations provide a rapid and convenient method for the prediction of thermophysical properties.^15^ The quality of these equations depends on the availability of accurate experimental and simulation data.^18−22^ For aqueous alkaline solutions, experimental data for self-diffusivities and solubilities of H_2_ and O_2_ at high concentrations (above 4 mol/kg), temperatures (323–373 K), and pressures (above 50 bar) is lacking, especially in the case of aqueous NaOH solutions.^14,27^ These temperatures (ca. 353 K) and concentrations (4–12 mol/kg electrolyte solution) are especially relevant for alkaline electrolyzers.^3,7,28−30^ Molecular simulations (i.e., molecular dynamics (MD) and Monte Carlo (MC)) can be used as a complementary approach to experiments^31^ to provide insight at conditions for which experimental data are limited and difficult to obtain due to high temperatures, pressures, and the corrosiveness of the solution (in case of strong alkaline solutions).

Molecular simulations of electrolyte systems can be studied using either ab initio simulations or force-field-based methods.^1,32,33^ Ab initio simulations have the potential to more accurately describe the structure and solvation of the ions,^32,34^ but these simulations are computationally expensive and are limited to systems comprising hundreds of atoms for time scales of the order of pico-seconds. To precisely calculate transport properties of fluids, long simulations of several nanoseconds are essential.^24,35^ To account for ion–ion and ion–water interactions at high electrolyte concentrations, water molecules need to be modeled explicitly,^36,37^ which makes the computations more costly. To overcome both the time and system-size restrictions of ab initio calculations, force-field-based methods are usually preferred for large-scale production of thermophysical data.

Force fields for aqueous electrolytes can be polarizable or nonpolarizable.^38−41^ The nonpolarizable TIP4P/2005 water model^42^ has proven to be quite suitable for predicting densities, viscosities, and self-diffusivities of water.^42−44^ In an attempt to model the effective charge screening that occurs in electrolyte solutions, ions are modeled as scaled charges in nonpolarizable force fields.^1^ Prior research has demonstrated that the use of scaled charges significantly helps in capturing the correct dynamics of ions.^38,45−47^ Scaled charge models for ions such as Na^+^, K^+^, and Cl^–^ have been developed by Zeron et al. (the so-called Madrid-2019 force field)^38,47^ and used in combination with the TIP4P/2005 water model.^38^ These force fields yield reasonable predictions for densities, dynamic viscosities, and self-diffusivities of aqueous electrolytes with a scaled charge of 0.85 for concentrations up to 4 mol/kg salt.^38^ However, the dynamic viscosities computed using the Madrid-2019 force field deviate from experiments at higher molalities. To address this, Vega and co-workers have developed a new force field called the Madrid-Transport with a scaled charge of 0.75.^47^ This force field can accurately predict dynamic viscosities of aqueous NaCl and KCl solutions up to their solubility limit.^47^ Despite the importance of alkaline systems, there is no Madrid-force field for OH^–^ to accurately predict densities and dynamic viscosities of aqueous NaOH and KOH systems. Existing OH^–^ force fields are often used to simulate the solvation energy^48−50^ and structure,^51−56^ and cannot be used directly in combination with the TIP4P/2005 water model and the Madrid-force fields^38,47^ for Na^+^ and K^+^ ions as they do not use scaled charges of -0.85 or -0.75.

Here, we propose several nonpolarizable two-site OH^–^ force fields with scaled charges of −0.85 and −0.75, respectively. One of the newly proposed OH^–^ force fields with a scaled charge of −0.75 yields accurate predictions for both densities and dynamic viscosities of aqueous NaOH and KOH solutions for concentrations ranging from 0 to 8 mol/kg, at temperatures ranging from 298 to 353 K. We use this force field to compute the self-diffusivities of H_2_ and O_2_ in aqueous NaOH and KOH solutions using MD. Solubilities of these gases as functions of concentrations, and temperatures, and pressures are computed using Continuous Fractional Component Monte Carlo (CFCMC) simulations.^57−59^ Our data, obtained from molecular simulations, are compared to available experimental data on H_2_ and O_2_ in KOH solutions. Our simulations can adequately describe the trends observed in experiments for variations in both concentration and temperature. The self-diffusivities and solubilities of H_2_ and O_2_ in NaOH and KOH solutions are then fitted to semiempirical engineering equations. These engineering equations can be used for process modeling, and for optimizing electrolyzers and fuel cells.^12^

This paper is organized as follows. In section 2, details on the force fields are provided, and the molecular simulation (MD and MC) techniques are explained. In section 3, force field optimization of OH^–^ is discussed, and the results for viscosities, H_2_ and O_2_ self-diffusivities, and solubilities at temperatures ranging from 298 to 353 K are provided. Our conclusions are summarized in section 4.

## 2 Methodology

### 2.1 Force Fields

The four-site TIP4P/2005 water model is used in all simulations.^42^ This model can accurately describe the densities and transport properties of pure H_2_O and of gases dissolved in H_2_O for a wide range of conditions.^31,42−44,60,61^ The two-site Bohn model^62^ is used for modeling O_2_. For H_2_, the single-site Vrabec model^63^ and the three-site Marx model^64^ are used. These force fields for H_2_ and O_2_ have shown to accurately describe gas diffusivities in pure water at various pressures and temperatures.^31^ The single-site H_2_ Vrabec model is less computationally demanding (no bonds or angles) than the three-site Marx model and yields similar self-diffusivities in pure TIP4P/2005 water (see Figure S1). This force field is used for computing self-diffusivities of H_2_ in NaOH and KOH solutions. The Marx model yields significantly more accurate H_2_ solubilities than the Vrabec model in pure TIP4P/2005 water (see Figure S1) and is used for computing H_2_ solubilities in NaOH and KOH solutions. For the K^+^ and Na^+^ ions, the Madrid-Transport (+0.75)^47^ and Madrid-2019 (+0.85)^38^ force fields are used (parameters listed in Table 1). For OH^–^, several force fields are proposed in this work. The details for OH^–^ force field are discussed in section 3.1. All force fields considered in this work are rigid. All interaction parameters for the TIP4P/2005 water, H_2_, and O_2_ models are provided in the Supporting Information (Tables S1–S3). The Lennard-Jones (LJ) and Coulombic interactions are considered for modeling the intermolecular interactions. The Lorentz–Berthelot mixing rules^65,66^ are applied with the exception of [Na/K – H_2_O] LJ interactions as specified in Table 1.

**Table 1:** Force Field Parameters for the Na^+^ and K^+^ Models Used (Madrid-2019^38^ and Madrid-Transport^47^)^a^

	Madrid-2019		Madrid-Transport	
	Na^+^	K^+^	Na^+^	K^+^
*q*_M_/[e]	0.85	0.85	0.75	0.75
ϵ_MM_/*k*_B_/[K]	177.08	238.83	177.08	238.83
σ_MM_/[Å]	2.21737	2.30140	2.21737	2.30140
ϵ_MO_W__/kB/[K]	95.42	168.43	95.42	168.43
σ_MO_W__/[Å]	2.60838	2.89040	2.38725	2.89540

a ϵ and σ are the Lennard-Jones parameters and *q* is the atomic partial charge. M refers to the Na^+^ or K^+^ atom. O_W_ refers to the O-atom of water (TIP4P/2005^42^ model). The Lorentz–Berthelot mixing rules^65,66^ are applied for all mixtures, with the exception of [Na/K – H_2_O] LJ interactions as specified in this table.

### 2.2 MD Simulations

MD simulations are carried out as implemented in the open-source Large-scale Atomic/Molecular Massively Parallel Simulator (LAMMPS).^67^ The Verlet algorithm,^68^ with a time step of 1 fs, is used for integrating the equations of motion. Periodic boundary conditions are imposed in all directions. For H_2_O, O_2_, and OH^–^, the SHAKE algorithm in LAMMPS^67,69^ is used to fix the bond lengths (and the bond angle of H_2_O). Analytic tail corrections for energies and pressures are applied to the LJ part of the potential. The cutoff radius for both LJ and Coulombic potentials is set to 10 Å. The particle–particle particle-mesh (PPPM)^66,70^ method is used for long-range electrostatic interactions with a relative error of 10^–5^.

The OCTP tool^71^ is used in LAMMPS to calculate the transport properties. The simulations are initially equilibrated in the *NPT* and *NVT* ensembles for a period of ca. 2 ns. Production runs (in *NVT*) of 10–50 ns are used to calculate dynamic viscosities and self-diffusivities. To obtain an ensemble mean and a standard deviation, each calculation is repeated 5 times with a different random seed for the initial velocity. The Nosé–Hoover thermostat and barostat^66,72,73^ are used, with a coupling constant of 100 and 1000 fs, respectively. The modifications of the Nosé–Hoover for rigid bodies, proposed by Kamberaj,^74^ are used in LAMMPS. The densities and transport properties are calculated in a simulation box containing 700 H_2_O molecules. The corresponding numbers of NaOH and KOH molecules, in combination with the respective molarities are provided in Tables S4 and S5. All initial configurations are created using the PACKMOL software.^75^ Two gas molecules (H_2_ or O_2_) (corresponding to infinite dilution) are used to calculate self-diffusivities of the gases in the aqueous NaOH and KOH solutions. All self-diffusivities, computed from mean-square displacements,^71^ are corrected for finite-size effects using the Yeh–Hummer equation:^76−79^
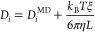
1where  and *D*_*i*_ denote the self-diffusivities calculated by MD and corrected for finite-size effects for species *i*, respectively, *k*_B_ is the Boltzmann constant, *T* is the temperature (in K), ξ is a dimensionless number equal to 2.837298 for a cubic simulation box, and *L* is the length of the simulation box.^76,78^ The dynamic viscosities (η), obtained from the MD simulations, do not have finite-size effects.^78,80−82^ To ensure no precipitation takes place and to calculate radial distribution functions, simulations are also carried out for a larger box size with 4200 H_2_O molecules for 10 ns.

### 2.3 CFCMC Simulations

The solubilities in this work are calculated using Henry coefficients (*H*).^83^ The Henry coefficients for H_2_ and O_2_ in aqueous NaOH and KOH solutions are computed using MC simulations in the Continuous Fractional Component^57−59^ isobaric–isothermal (CFCNPT) ensemble. All MC simulations are carried out using the open-source Brick-CFCMC software.^57,84,85^ All molecules are considered as rigid, and only intermolecular LJ and Coulombic interactions are considered. A cutoff radius of 10 Å is used for both the LJ and Coulombic interactions. The Ewald summation with a relative precision of 10^–6^ is used for the electrostatics. Analytic tail corrections for energies and pressures are applied to the LJ part of the potential.^66^ Periodic boundary conditions are imposed in all directions. All MC simulations contained 300 water molecules. For all the compositions considered, the corresponding numbers of NaOH and KOH molecules and the respective molarities are provided in Table S4 and S5. Simulations are carried out at temperatures of 298, 323, 333, 343, and 353 K, at H_2_ and O_2_ pressures of 1, 50, and 100 bar.

To calculate the excess chemical potentials and solubilities of H_2_ and O_2_, “fractional” molecules are introduced. In contrast to “whole” or normal molecules, the interactions of “fractional” molecules with other molecules are scaled with a continuous order parameter λ (in the range of [0, 1]):^57,86^ λ = 0 indicates no interactions between the fractional and whole molecules (ideal gas), while λ = 1 indicates full interactions, corresponding to a “whole” or normal/unscaled molecule. For more details regarding the scaling of the interactions of fractional molecules the reader is referred to refs (87−89). A single fractional molecule of H_2_ (and O_2_) is used to calculate the excess chemical potentials of the respective molecules in the solution. All other molecules in the simulation are whole molecules. The Wang–Landau algorithm^90,91^ is used to construct a biasing weight function for λ (*W*(λ)). The biasing weight function helps in overcoming possible energy barriers in λ-space, to ensure a flat observed probability distribution.^83^ 100 bins are used to obtain a histogram of λ values, thereby computing the probability of occurrence for each λ value. The Boltzmann average of any parameter (*A*) can be computed using^83^
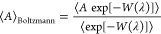
2The infinite dilution excess chemical potential (μ^ex,*∞*^) can be related to the Boltzmann sampled probability distribution of λ (*p*(λ)) using^57,83^
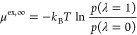
3where *p*(λ = 1) and *p*(λ = 0) are the Boltzmann sampled probability distribution of λ at 1 and 0, respectively. The molarity based Henry coefficient (*H*) is defined as^83^
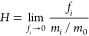
4in which *f*_*i*_ is the fugacity of a solute in the gas phase, *m*_*i*_ is the molarity of the gas in the solution (mol/L), and *m*_0_ is set to 1 mol/L. The infinite dilution excess chemical potential of H_2_ and O_2_ can be related to the molarity based Henry coefficient using^83^
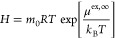
5where *R* is the universal gas constant. For all simulations, 4 × 10^5^ equilibration cycles are carried out followed by 4 × 10^5^ production cycles. A cycle refers to *N* number of trial moves, with *N* corresponding to the total number of molecules, with a minimum of 20. Trial moves are selected with the following probabilities: 1% volume changes, 35% translations, 29% rotations, 25% λ changes, and 10% reinsertions of the fractional molecules at random locations inside the simulation box. The maximum displacements for volume changes, molecule translations, rotations, and λ changes are adjusted to obtain ca. 50% acceptance of trial moves. For each condition (concentration, temperature, pressure), 20 independent simulations are performed. The final Boltzmann probability distributions of λ are averaged in blocks of 4 to obtain 5 independent averaged distributions. For all averaged distributions, the excess chemical potentials and Henry coefficients are calculated to obtain a mean value and the standard deviation. All the raw data for the MD and MC simulations are shown in Tables S6–S11.

## 3 Results and Discussion

### 3.1 Force Field Optimization

To construct accurate models for aqueous NaOH and KOH solutions, four different two-site OH^–^ (i.e., O^δ−^ and H^δ+^) force fields are considered (FF1–FF4) combined with the TIP4P/2005 water^42^ and the Madrid-2019^38^ or Madrid-Transport^47^ force fields for Na^+^ and K^+^. These force fields and their corresponding parameters are listed in Table 2. For all OH^–^ models, the O–H bond length is set to 0.98 Å, similar to the works of refs (52) and (53). FF1, FF3, and FF4 have a total scaled charge (*q*_OH_) of −0.75 on OH^–^, while FF2 has a total scaled charge of −0.85. These force fields are used in combination with the Madrid-Transport (+0.75)^47^ and Madrid-2019 (+0.85)^38^ Na^+^ and K^+^ models such that the total charge of NaOH and KOH clusters becomes 0. The charge of OH^–^ is distributed on the O (*q*_O_) and H (*q*_H_) atom. For FF1 and FF2, the charges on the O and H atoms have the same ratios as in the work by Botti et al. on the structure of concentrated NaOH solutions.^52^ The charge distributions of the FF3 and FF4 models are based on Quantum Theory of Atoms in Molecules (QTAIM) calculations for OH^–^, which have indicated that the O atom can have an unscaled charge of −1.4 to −1.3.^92^ For this reason, for the FF3 and FF4 models, the charge on the O atom are set to −1.4 × 0.75 and −1.3 × 0.75, respectively. The charge on the H atom (*q*_H_) is set such that *q*_O_ + *q*_H_ = *q*_OH_. For each force field, the Lennard-Jones σ parameter of the O atom (σ_OO_) is adjusted based on the experimental densities of aqueous NaOH and KOH solutions.^10,93,94^

**Table 2:** Force Field Parameters for OH^–^a

model	*q*_O_/[e]	*q*_H_/[e]	*q*_OH_	σ_OO_/[Å]	ϵ_OO_/*k*_B_/[K]	σ_HH_/[Å]	ϵ_HH_/*k*_B_/[K]
FF1	–1.2181	+0.4681	–0.75	3.65	30.19	1.443	22.13
FF2	–1.3805	+0.5305	–0.85	3.85	30.19	1.443	22.13
FF3	–1.0500	+0.3000	–0.75	3.55	30.19	1.443	22.13
FF4	–0.9750	+0.2250	–0.75	3.45	30.19	1.443	22.13

a The bond length of O–H is set to 0.98 Å. For all models, the sigma for H (σ_HH_) is set to 1.443 Å.^52^ The Lennard-Jones ϵ parameters for O and H (ϵ_OO_/*k*_B_, ϵ_HH_/*k*_B_) are based on refs (52) and (56) and are set to 30.19 and 22.13 K, respectively, for all the models. The FF1 force field for OH^–^ is recommended.

**Figure 1:**
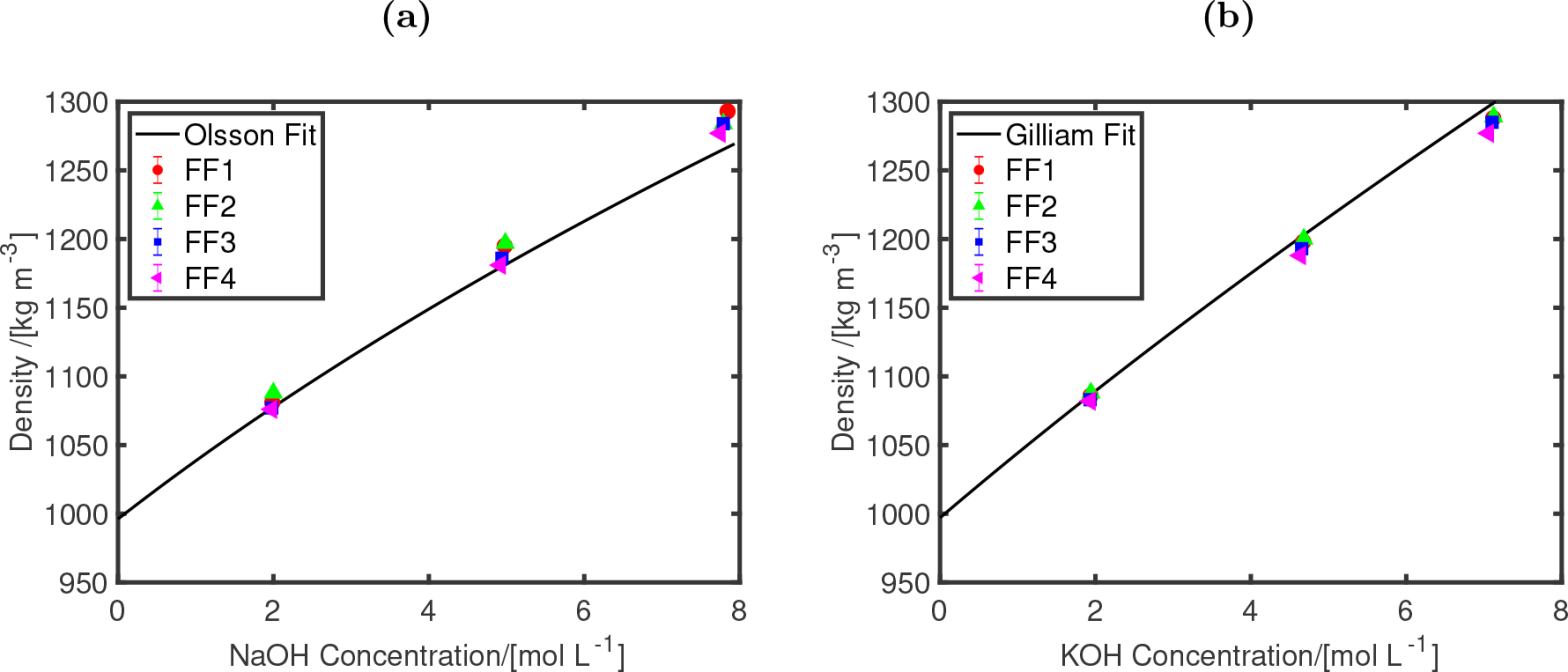
Densities at 298 K and 1 bar as functions of the electrolyte concentrations for (a) NaOH and (b) KOH. Four different OH^–^ force fields are considered (FF1–FF4) and compared to experimental fits (shown as lines) of Olsson^94^ (for NaOH) and Gillam^10,93^ (for KOH). The different parameters used for all the force fields are listed in Table 2.

Figure 1 shows the variation of densities as functions of electrolyte concentrations for both NaOH and KOH. By adjusting the value of σ_OO_, it is possible to obtain an excellent agreement for all the different models. All the densities obtained deviate less than 2% from experimental fits found in literature. A larger negative charge on O (*q*_O_) results in a larger optimum σ_OO_ parameter, to counteract the strong attractive Coulombic interactions. The experimental fits of Olsson^94^ (for densities and viscosities of aqueous NaOH), Gilliam^93^ (densities of aqueous KOH), and Guo^95^ (viscosities of aqueous KOH) are used and shown as lines in Figure 1.

**Figure 2:**
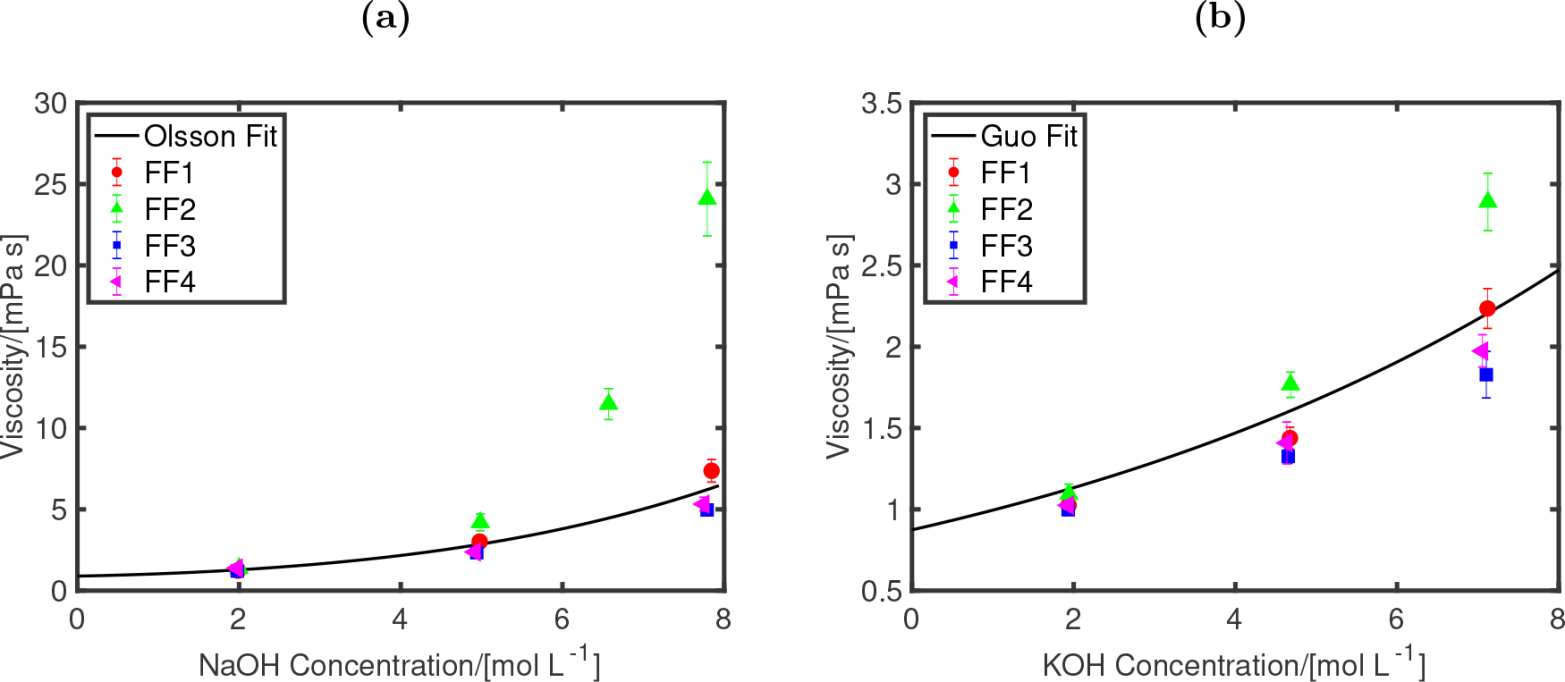
Dynamic viscosities at 298 K and 1 bar as functions of the electrolyte concentrations for (a) NaOH, and (b) KOH. Four different OH^–^ force fields are considered (FF1–FF4) and compared to experimental fits (shown as lines) of Olsson^94^ (for NaOH) and Guo^95^ (for KOH). The different parameters used for all the force fields are listed in Table 2.

The dynamic viscosities of aqueous NaOH and KOH solutions calculated using FF1–FF4 are shown in Figure 2. It can be observed that the choice of the total charge (*q*_OH_), and the resulting σ_OO_ has a significant influence on the viscosities, especially at higher concentrations in which the influence of ion–ion interactions become more important. The influence of ion size on the viscosities and densities is shown in Figure S2. In case of FF2 (with *q*_OH_ = −0.85), the dynamic viscosity is overestimated by more than a factor 3 compared to the experimental fit for the highest concentration of NaOH. For aqueous KOH, the FF2 model overestimates the dynamic viscosity by around 40% at the highest concentration of KOH. The FF1, FF3, and FF4 models with *q*_OH_ = −0.75 show a much better agreement with the experimental fit. The findings of the Madrid-Transport model for aqueous NaCl and KCl solutions^47^ also show that a scaled charge of 0.75 leads to better predictions of transport properties (especially at concentrations above 4 mol/kg salt) compared to a scaled charge of 0.85. Overall, the FF1 model shows the best agreement with the experimental viscosities and densities. For this reason, only the results of the FF1 model will be used and discussed further in this work.

**Figure 3:**
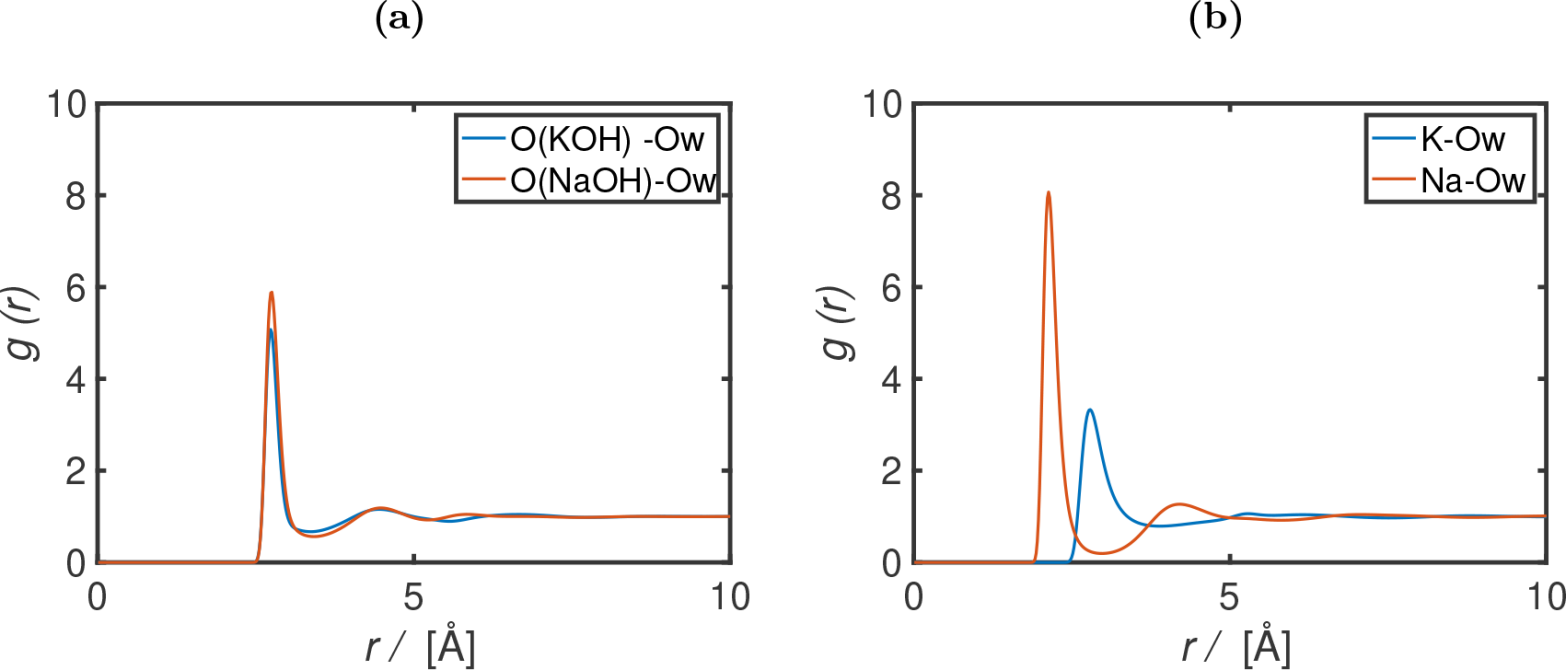
Radial distribution functions (*g*(*r*)) for (a) O(KOH)–O_W_ (O of water) and O(NaOH)–O_w_ and (b) K^+^(KOH)–O_W_ and Na^+^(NaOH)–O_W_, as a function of radial distance *r* (Å), at 298 K, 1 bar, and a concentration of 5 mol/kg (corresponding to a molarity of 4.98 mol/L for NaOH, and 4.68 mol/L for KOH). The FF1 OH^–^ model, in combination with the TIP4P/2005 water model^42^ and the Madrid-Transport Na^+^ and K^+^ models,^47^ are used for the MD simulations.

The radial distribution functions (RDFs) for anion–O_W_ (O of water) and cation–O_W_ are shown in Figure 3. The RDFs for the anion–anion, anion–cation, and cation–cations are shown in Figure S3. Based on the RDFs, the hydration numbers (*n*_hyd_) are calculated using^38^
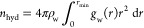
6where *g*_w_ is the anion/cation–O_W_ RDF, *r* is the radial distance, *r*_min_ is the position of the first minimum in the RDF, and ρ_w_ is the number density of water in the solution. Our results show a first peak at approximately 2.13 and 2.79 Å for Na^+^–O_W_ and K^+^–O_W_, respectively. The cation hydration numbers are 4.9 and 7.2 for Na^+^ and K^+^, respectively, at a molality of 5 mol/kg (corresponding to a molarity of 4.98 mol/L for NaOH, and 4.68 mol/L for KOH). Crystallization of ions is not observed for all our MD simulations of 10–50 ns based on the RDFs. Experimental and simulation results in literature suggest a first RDF peak at approximately 2.4–2.5 Å^33,52,55^ for Na^+^–O_W_ and a peak at approximately 2.7–2.8 Å for K^+^–O_W_.^53^ The reported hydration numbers (in the first shell) are in the ranges of 4–8 and 6–8 for Na^+^ and K^+^, respectively.^96^ For OH^–^, the results show a first peak at approximately 2.75 Å for OH^–^–O_W_, with hydration numbers of 4.8 and 5.9 for KOH and NaOH, respectively, at a molality of 5 mol/kg. Other molecular simulations in literature report a first peak ranging from 2.3 to 2.7 Å for the first OH^–^–O_W_ peak.^33,52,55^ The combined Car–Parrinello MD and X-ray diffraction studies of Megyes et al. for aqueous NaOH report a OH^–^–O_W_ distance ranging from 2.65 to 2.70 Å, with hydration numbers ranging from 3 to 5.^33^ Overall, our force field results show agreement with other studies, albeit slightly overpredicting the first OH^–^–O_W_ peak and the hydration.

The self-diffusivities of NaOH and KOH are listed in Table 3. Even though for Na^+^ and K^+^ the self-diffusivities at infinite dilution are close, this is not the case for OH^–^ (underestimated by a factor ca. 5). For reasonable values of σ_OO_, ϵ_OO_, and *q*_O_, we could not obtain OH^–^ self-diffusivities close to the values reported by experiments^97^ without causing significant deviations from experimental densities and viscosities. This result is expected as classical OH^–^ models cannot capture the details of the solvation of OH^–^ in water and the proton transfer mechanism, which lead to anomalously high OH^–^ mobilities as discussed by Tuckerman et al.^34,98^ As such, our model, similarly to other classical force fields, is not suitable for predicting OH^–^ diffusivities of NaOH and KOH. Since electrical conductivities vastly depend on the mobility of the OH^–^ ions in the solution, the new OH^–^ model presented here is unable to accurately predict electrical conductivities of aqueous NaOH and KOH solutions. Although our classical force field cannot capture the proton transfer mechanism, it can correctly predict the dynamic viscosities of the electrolyte solutions. As the aim of this study is to study the transport properties and solubilities of H_2_ and O_2_ gas in aqueous NaOH and KOH electrolytes, correct predictions of densities and viscosities are sufficient. Developing an OH^–^ force field by taking into account the proton transfer mechanism and accurate OH^–^ mobilities is beyond the scope of this work as quantum mechanical based force fields will be required.

**Table 3:** Finite Size-Corrected (Using eq 1) Self-Diffusivities of Cations (*D*_cation_) (Na^+^, K^+^) and OH^–^ at Different Molalities of 1.99 and 0.48 mol/kg Calculated Using MD^a^

	*D*_cation_/[10^–9^ m^2^ s^–1^]			*D*_OH^–^_/[10^–9^ m^2^ s^–1^]		
	MD		expt	MD		expt
molality (mol/kg)	1.99	0.48	0	1.99	0.48	0
NaOH	1.02	1.36	1.33	0.90	1.17	5.27
KOH	1.59	1.95	1.96	1.09	1.23	5.27

a A comparison is made with experimental diffusion coefficients at infinite dilution of ions.^97^ The FF1 OH^–^ model, in combination with the TIP4P/2005 water model^42^ and the Madrid-Transport Na^+^ and K^+^ models,^47^ are used for the MD simulations.

### 3.2 Densities and Viscosities

**Figure 4:**
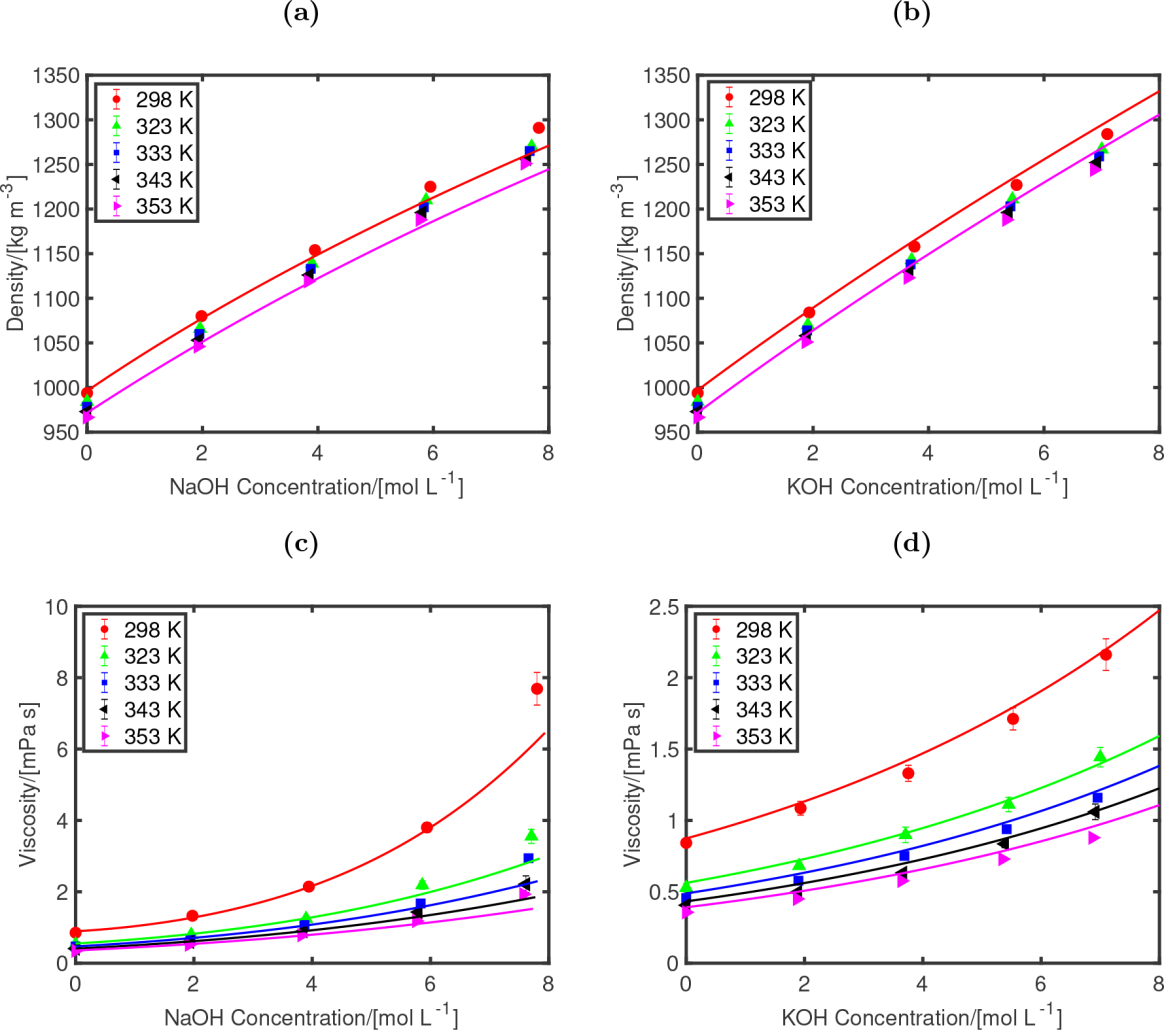
Densities (a, b) and dynamic viscosities (c, d) as functions of concentrations (mol/L) for aqueous NaOH (a, c) and KOH (b, d) at 1 bar. The simulations results at temperatures 298 (red), 323 (green), 333 (blue), 343 (black), and 353 (purple) K are shown. The lines represent experimental correlations. For densities, the Olsson^94^ and Gilliam correlations^93^ at 298 (red) and 353 (purple) K are shown. For viscosities, the Olsson^94^ (NaOH) and Guo^95^ (KOH) correlations are plotted for all temperatures with the same color scheme as the simulation points. The FF1 OH^–^ model, in combination with the TIP4P/2005 water model^42^ and the Madrid-Transport Na^+^ and K^+^ models,^47^ is used for the MD simulations.

It is important to show that the NaOH and KOH models (FF1 OH^–^ model, and the Madrid-Transport models of Na^+^, and K^+^)^47^ can accurately predict the temperature-dependence of densities and viscosities. Figure 4 shows the densities and viscosities at different temperatures for both NaOH and KOH solutions. The agreement between MD simulations and experimental fits is excellent for aqueous KOH. For aqueous NaOH solutions, the results of densities are overestimated by ca. 2% and for dynamic viscosities by ca. 20% at the highest concentration (molality 8 mol/kg). Despite this, the trends of densities and viscosities for variations of electrolyte concentration and temperature are well-predicted by the MD simulations using the new force fields. Densities and viscosities show a much weaker dependence on pressure (in the range of 1 to 100 bar) compared to temperature (in the range of 298 to 353 K) due to the incompressibility of the liquid phase. The variations of densities and viscosities as a function of pressure are shown in Figure S4.

### 3.3 Self-Diffusivities of H_2_ and O_2_ in Aqueous NaOH and KOH

**Figure 5:**
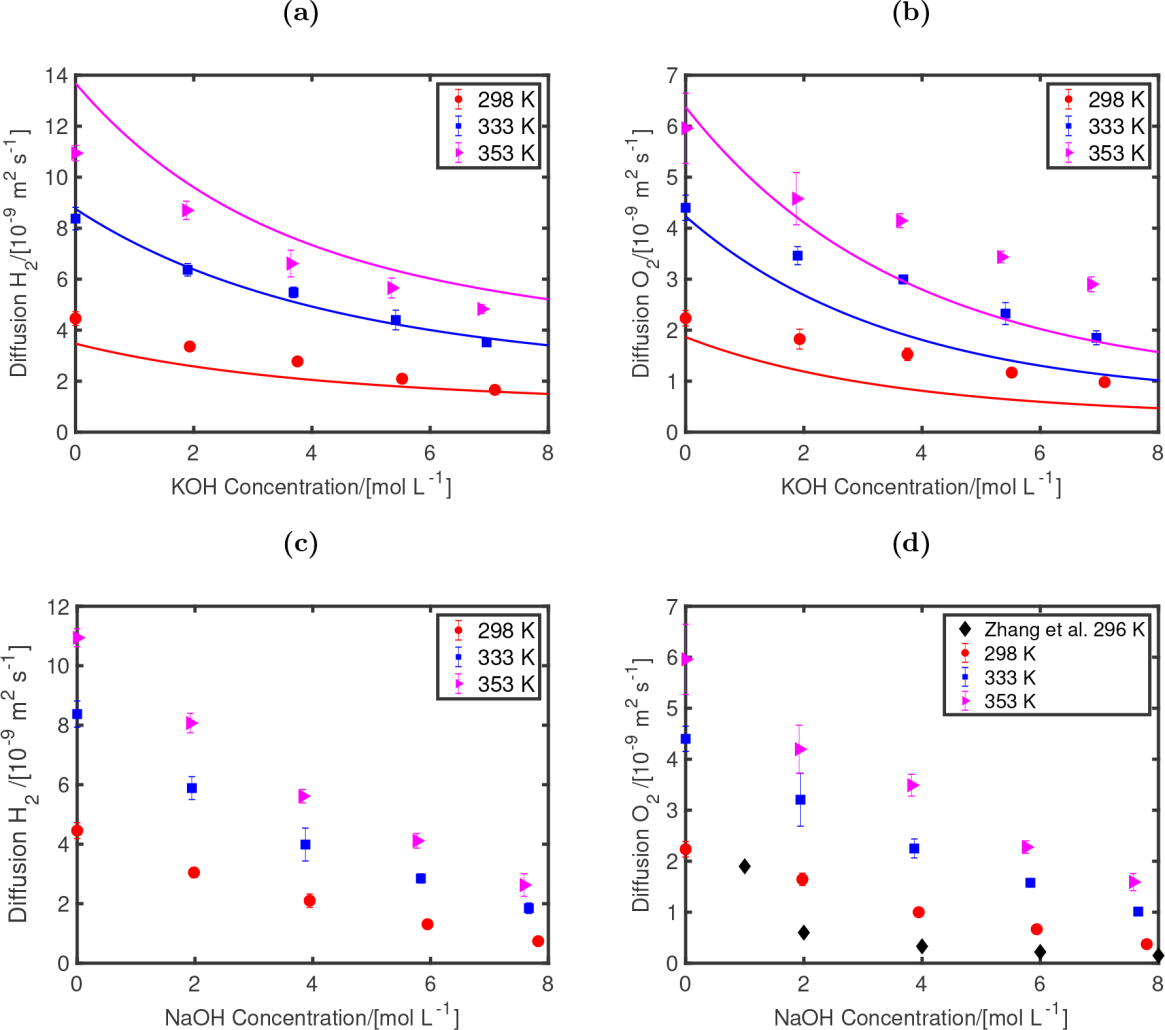
H_2_ (a, c) and O_2_ (b, d) self-diffusivities as functions of KOH (a, b) and NaOH (c, d) concentrations at different temperatures (298, 333, and 353) K at 1 bar. For diffusivities of H_2_ and O_2_ in the KOH solution, the experimental data of Tham et al.^27,99^ at 298 (red), 333 (blue), and 353 K (purple) are fitted using eq 4 and shown in (a) and (b) as lines. The fitting coefficients of these data are shown in Table 4. The experimental diffusivities of O_2_ in NaOH solution at 296 K (black) provided by Zhang et al.^14^ are plotted as points. The FF1 OH^–^ model, in combination with the TIP4P/2005 water model,^42^ the Bohn O_2_ model,^62^ the Vrabec H_2_ model,^63^ and the Madrid-Transport Na^+^ and K^+^ models,^47^ is used for the MD simulations.

The finite size-corrected self-diffusivities (using eq 1) of H_2_ and O_2_ in aqueous NaOH and KOH solutions calculated using MD simulations at various temperatures are shown in Figure 5. The results obtained by our MD simulations for the KOH solution are compared to the experimental data of Tham et al.^27,99^ at different temperatures, i.e., 298, 333, and 353 K. For H_2_ self-diffusivities, our results are in quantitative agreement with the results of Tham et al.^27,99^ The increase in H_2_ and O_2_ diffusivities at higher temperatures are well-predicted. These trends are linked to the decrease of the dynamic viscosities of the solutions, which the MD simulations capture correctly. In our simulations for O_2_, the decay in the self-diffusivities with respect to variations of KOH concentrations are underpredicted with respect to the experimental data. Zhang et al.^14^ report experimental O_2_ diffusivities in aqueous NaOH at 296 K. Although the results of Zhang et al.^14^ for O_2_ diffusivity at 1 mol/L NaOH is in agreement to ours, at 2 mol/L their results show a sharp decrease of the O_2_ diffusivities by approximately a factor 1/3 with respect to diffusivities at 1 mol/L NaOH.^14^ This sharp decline is not observed in our calculations. However, the current force field models have managed to qualitatively predict the trends for a wide concentration (0–8 mol/kg) and temperature (298–353 K) range. For H_2_ self-diffusivities in aqueous NaOH no experimental data at these different temperatures are found. Thus, our simulations serve as a first prediction for these data.

The simulations results (at 298, 323, 333, 343, and 353 K) in this work (shown in Figure S5), and the experimental data of Tham et al. (at 298, 333, and 353 K)^27,99^ are fitted to an engineering equation with an Arrhenius-inspired term for temperature variations:
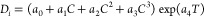
7where *D*_*i*_ is the self-diffusivity of H_2_ and O_2_ in NaOH and KOH solutions, *a*_0_–*a*_4_ are fitting constants, *C* is the electrolyte concentration (in mol/L), and *T* is the temperature (in K). All fitting parameters for H_2_ and O_2_ in the aqueous NaOH and KOH solutions are listed in Table 4. Equation 7 provides an excellent fit for both the simulation results found in this work and the experimental data of Tham et al.^27^ as shown in Figure S5.

**Table 4:** Fitting Parameters for eq 7 for H_2_ and O_2_ Self-Diffusivities in Aqueous NaOH and KOH Solutions^a^

	*a*_0_	*a*_1_	*a*_2_	*a*_3_	*a*_4_
H_2_–KOH (expt)	0.4066	–0.5903	0.4748	–0.1421	2.288
O_2_–KOH (expt)	0.2625	–0.5124	0.4345	–0.1278	2.201
H_2_–KOH (MD)	3.844	–5.006	3.686	–1.511	1.606
O_2_–KOH (MD)	1.511	–2.092	2.483	–1.743	1.701
H_2_–NaOH (MD)	3.344	–5.725	4.649	–2.103	1.648
O_2_–NaOH (MD)	1.313	–2.105	1.604	–0.7482	1.743

a The values for *a*_0_ (in units of 10^–^^1^^1^ m^2^/s), *a*_1_ (in units of 10^–12^ m^2^/s (L/mol)), *a*_2_ (in units of 10^–13^ m^2^/s (L/mol)^2^), *a*_3_ (in units of 10^–14^ m^2^/s (L/mol)^3^), and *a*_4_ (in units of 10^–^^2^ K^–^^1^) are shown for both the MD simulations obtained in this work (range of validity: 0-8 mol/L, 298-353 K), and the experimental work of Tham et al. (at 298, 333, and 353 K) for H_2_ and O_2_ diffusion coefficient in KOH solutions (range of validity: 0–14 mol/L). The FF1 OH^–^ model, in combination with the TIP4P/2005 water model, the Bohn O_2_ model, the Vrabec H_2_ model, and the Madrid-Transport Na^+^, and K^+^ models is used for the MD simulations.

### 3.4 Solubilities of H_2_ and O_2_ in Aqueous NaOH and KOH

**Figure 6:**
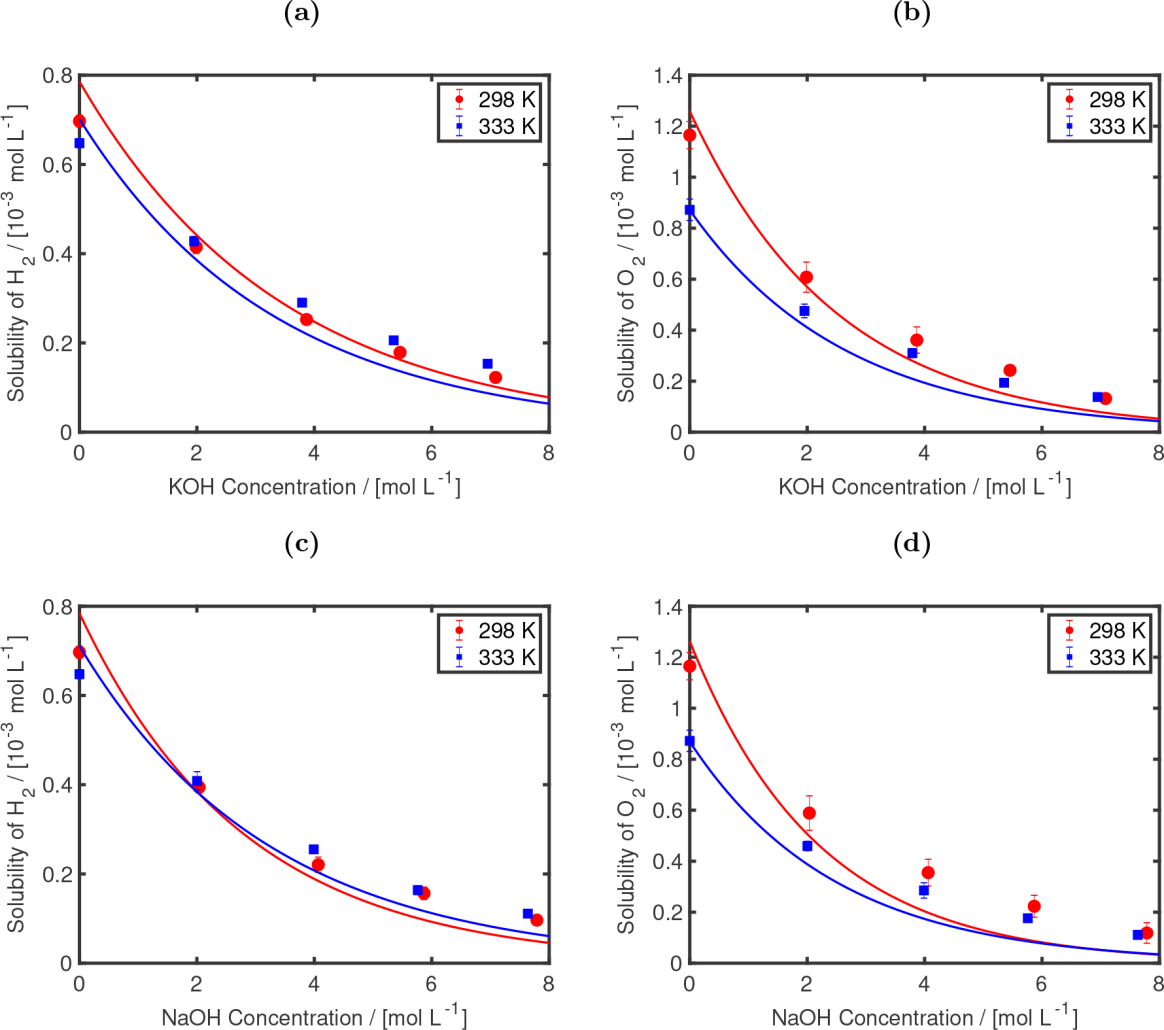
H_2_ (a, c) and O_2_ (b, d) solubilities as functions of KOH (a, b) and NaOH (c, d) concentrations at different temperatures of 298, and 333 K at 1 bar. For solubilities of H_2_ and O_2_ in KOH solutions, the experimental data of Walker et al.^99^ at 298 (red), and 333 K (blue) is fitted using eq 8 and shown in (a) and (b) as lines. The fitting coefficients are shown in Table 5. For H_2_ and O_2_ solubilities in NaOH solutions, the Sechenov model^100,101^ (using the parameters provided by Weisenberger et al.^101^), and the experimental solubilities in pure water^99^ are used to obtain the experimental fits, which are shown as lines. The FF1 OH^–^ model, in combination with the TIP4P/2005 water model,^42^ the Bohn O_2_ model,^62^ the Marx H_2_ model,^64^ and the Madrid-Transport Na^+^ and K^+^ models,^47^ is used for the MC simulations.

In Figure 6, the H_2_ and O_2_ solubilities obtained using CFCMC calculations are shown as functions of NaOH and KOH concentrations. In this figure, only the results at 298 and 333 K are shown as solubilities (especially at higher electrolyte concentrations) vary only weakly in the temperature range of 298–353 K. The solubilities of H_2_ and O_2_ at 298, 323, 333, 343, and 353 K are shown in Figure S7.

As a comparison the experimental data provided by Walker et al.^99^ on the solubilities of H_2_ and O_2_ in aqueous KOH are fitted and plotted in Figure 6a,b. This experimental data are also in agreement with the experiments of Davis et al.^102^ for O_2_ solubilities (at 298 and 333 K) and with the Sechenov model.^101^ The Sechenov model^100^ (with the parameters provided by Weisenberger et al.)^101^ is an empirical model, which predicts the salting out effect^103,104^ at different temperatures (273–363 K) and electrolyte concentrations.^101^ For NaOH, our data are compared to the Sechenov model as direct experimental data at these two temperatures are not available. Zhang et al.^14^ report solubilities of O_2_ in aqueous NaOH at 296 K. Our simulations show agreement with data and experimental fits for both H_2_ and O_2_. Both the salting out phenomena and the temperature trends are captured by our simulations. At low electrolyte concentrations (below 2 mol/L), increasing the temperature from 298 to 333 K leads to slightly lower H_2_ and O_2_ solubilities. At higher molarities, the solubilities become less dependent on the temperature and the concentration of the salts dominate the solubilities. The simulations results and experimental data of Walker et al.^99^ for H_2_ and O_2_ solubilities in aqueous KOH and NaOH are fitted to a Sechenov-based^101^ engineering equation:
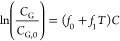
8where *C*_G_ and *C*_G,0_ are the solubility of the gas in the electrolyte and pure water at 1 bar, respectively. *f*_0_ and *f*_1_ are fitting constants. The temperature dependence of the parameter *C*_G,0_ can be fitted as
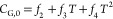
9where *f*_2_–*f*_4_ are additional fitting parameters. The optimized fitting parameters for MC simulations in this work and the experimental data of Walker et al. for H_2_ and O_2_ solubilities are shown in Table 5. Equation 8 provides an excellent fit for both the simulation results found in this work and the experimental data present in the literature, as shown in Figure S7.

**Table 5:** Fitting Parameters for eq 8 for the H_2_ and O_2_ Solubilities (mol/L) in NaOH and KOH Solution^a^

	*f*_0_	*f*_1_	*f*_2_	*f*_3_	*f*_4_
H_2_–KOH (expt)	–1.944	–3.167	9.517	–5.337	8.078
O_2_–KOH (expt)	–5.712	5.854	16.961	–8.993	12.494
H_2_–KOH (MC)	–5.468	10.077	4.874	–2.526	3.773
O_2_–KOH (MC)	–5.670	8.889	13.935	–7.331	10.218
H_2_–NaOH (MC)	–4.749	7.241	4.874	–2.526	3.773
O_2_–NaOH (MC)	–4.093	4.057	13.935	–7.331	10.218

a The values for *f*_0_ (10^–1^ (L/mol)), *f*_1_ (10^–4^ (L/mol) K^–1^), *f*_2_ (10^–3^ (mol/L)), *f*_3_ (10^–5^ (mol/L) K^–1^), and *f*_4_ (10^–8^ (mol/L) K^–1^) are shown for both the MC simulations obtained in this work (range of validity: 0–8 mol/L, 298–353 K), and the experimental work of Walker et al. (at 298, 333, and 353 K) for H_2_ and O_2_ solubilities in KOH solutions (range of validity: 0–14 mol/L). The FF1 OH– model, in combination with the TIP4P/2005 water model, the Bohn O_2_ model, the Marx H_2_ model, and the Madrid-Transport Na^+^ and K^+^ models, is used for the MC simulations.

## 4 Conclusions

The self-diffusivities and solubilities of H_2_ and O_2_ in aqueous NaOH and KOH solutions are modeled using MD and CFCMC simulations. A new two-site nonpolarizable OH^–^ force field (FF1 model) is proposed with a scaled charge of −0.75, which matches with the TIP4P/2005 water and the Madrid-Transport models for Na^+^ and K^+^. Although our classical force field cannot capture the proton transfer mechanism, which influences the OH^–^ diffusivities, it can predict the densities, dynamic viscosities, and the salting out of H_2_ and O_2_ in aqueous NaOH and KOH solutions. Excellent agreement is observed between simulation and experimental data for both densities and dynamic viscosities of NaOH and KOH for a concentration range of 0–6 mol/kg and a temperature range of 298–353 K. This model is used to generate self-diffusivity and solubility data for H_2_ and O_2_ in aqueous NaOH and KOH solutions for a temperature range of 298–353 K and a concentration range of 0–8 mol/kg. The computed data and existing experimental results are used to fit engineering equations. The obtained data and engineering equations can be used for process modeling and optimizing electrolyzers and fuel cells.

## Supporting Information Available

Comparison of the Marx^64^ and Vrabec^63^ H_2_ force fields for the prediction of H_2_ self-diffusivities (finite-size corrected^78^) and solubilities in pure TIP4P/2005 water; influence of OH^–^ ion size on densities, and viscosities at 1 bar and 298 K; radial distribution functions (RDFs) of cation–anion, cation–anion, and anion–anion of NaOH and KOH solutions at 1 bar and 298 K; variation of densities and dynamic viscosities for a pressure range of 1–100 bar at 298 K for different concentrations of aqueous NaOH and KOH; engineering fits for experimental and simulation data for diffusivities of H_2_ and O_2_ in aqueous NaOH and KOH solutions; solubilities of H_2_ and O_2_ for a pressure range of 1–100 bar at 298 K in pure water calculated using Monte Carlo simulations; engineering fits for experimental and simulation data for solubilities of H_2_ and O_2_ in aqueous NaOH and KOH solutions; force field details for TIP4P/2005 water,^42^ Vrabec^63^ H_2_, Marx^64^ H_2_, and Bohn^62^ O_2_;^38^ number of Na^+^ and K^+^ ions used for molecular dynamics and Monte Carlo simulations and the respective electrolyte weight percentages (wt %), molalities, and molarities (at 298 K and 1 bar); raw data for densities, viscosities, and self-diffusivities of H_2_ and O_2_ at various temperatures and concentrations of aqueous KOH at 1 and 100 bar; raw data for densities, viscosities, and self-diffusivities of H_2_ and O_2_ at various temperatures and concentrations of aqueous NaOH at 1 and 100 bar; raw data for excess chemical potentials, Henry coefficients, and solubilities of H_2_ and O_2_ at various temperatures and concentrations of aqueous KOH and NaOH at 1 bar (PDF)

## References

[ref1] Panagiotopoulos A. Z. Simulations of activities, solubilities, transport properties, and nucleation rates for aqueous electrolyte solutions. J. Chem. Phys. 2020, 153, 010903 10.1063/5.0012102. 32640801

[ref2] Hellström M.; Behler J. Structure of aqueous NaOH solutions: insights from neural-network-based molecular dynamics simulations. Phys. Chem. Chem. Phys. 2017, 19, 82–96. 10.1039/C6CP06547C. 27805193

[ref3] Bodner M.; Hofer A.; Hacker V. H_2_ generation from alkaline electrolyzer. Wiley Interdisciplinary Reviews: Energy and Environment 2015, 4, 365–381. 10.1002/wene.150.

[ref4] David M.; Ocampo-Martínez C.; Sánchez-Peña R. Advances in alkaline water electrolyzers: A review. Journal of Energy Storage 2019, 23, 392–403. 10.1016/j.est.2019.03.001.

[ref5] Solovey V.; Shevchenko A.; Zipunnikov M.; Kotenko A.; Khiem N. T.; Tri B. D.; Hai T. T. Development of high pressure membraneless alkaline electrolyzer. Int. J. Hydrogen Energy 2022, 47, 6975–6985. 10.1016/j.ijhydene.2021.01.209.

[ref6] Ulleberg Ø. Modeling of advanced alkaline electrolyzers: a system simulation approach. Int. J. Hydrogen Energy 2003, 28, 21–33. 10.1016/S0360-3199(02)00033-2.

[ref7] Merle G.; Wessling M.; Nijmeijer K. Anion exchange membranes for alkaline fuel cells: A review. J. Membr. Sci. 2011, 377, 1–35. 10.1016/j.memsci.2011.04.043.

[ref8] Solubility table of compounds in water at temperature. https://www.sigmaaldrich.com/NL/en/support/calculators-and-apps/solubility-table-compounds-water-temperature (Accessed Sep. 6, 2022).

[ref9] Potassium hydroxide. https://webwiser.nlm.nih.gov/substance?substanceId=401& (Accessed Sep. 6, 2022).

[ref10] Le Bideau D.; Mandin P.; Benbouzid M.; Kim M.; Sellier M. Review of necessary thermophysical properties and their sensivities with temperature and electrolyte mass fractions for alkaline water electrolysis multiphysics modelling. Int. J. Hydrogen Energy 2019, 44, 4553–4569. 10.1016/j.ijhydene.2018.12.222.

[ref11] Zarghami A.; Deen N.; Vreman A. CFD modeling of multiphase flow in an alkaline water electrolyzer. Chem. Eng. Sci. 2020, 227, 115926 10.1016/j.ces.2020.115926.

[ref12] Haug P.; Kreitz B.; Koj M.; Turek T. Process modelling of an alkaline water electrolyzer. Int. J. Hydrogen Energy 2017, 42, 15689–15707. 10.1016/j.ijhydene.2017.05.031.

[ref13] Haug P.; Koj M.; Turek T. Influence of process conditions on gas purity in alkaline water electrolysis. Int. J. Hydrogen Energy 2017, 42, 9406–9418. 10.1016/j.ijhydene.2016.12.111.

[ref14] Zhang C.; Fan F.-R. F.; Bard A. J. Electrochemistry of oxygen in concentrated NaOH solutions: solubility, diffusion coefficients, and superoxide formation. J. Am. Chem. Soc. 2009, 131, 177–181. 10.1021/ja8064254. 19063634

[ref15] Rowland D.; Königsberger E.; Hefter G.; May P. M. Aqueous electrolyte solution modelling: Some limitations of the Pitzer equations. Appl. Geochem. 2015, 55, 170–183. 10.1016/j.apgeochem.2014.09.021.

[ref16] Kontogeorgis G. M.; Maribo-Mogensen B.; Thomsen K. The Debye-Hückel theory and its importance in modeling electrolyte solutions. Fluid Phase Equilib. 2018, 462, 130–152. 10.1016/j.fluid.2018.01.004.

[ref17] Walker P. J.; Liang X.; Kontogeorgis G. M. Importance of the Relative Static Permittivity in electrolyte SAFT-VR Mie Equations of State. Fluid Phase Equilib. 2022, 551, 113256 10.1016/j.fluid.2021.113256.

[ref18] Costa Reis M. Current Trends in Predictive Methods and Electrolyte Equations of State. ACS Omega 2022, 7, 16847 10.1021/acsomega.2c00168. 35647467 PMC9134406

[ref19] Maribo-Mogensen B.; Thomsen K.; Kontogeorgis G. M. An electrolyte CPA equation of state for mixed solvent electrolytes. AIChE J. 2015, 61, 2933–2950. 10.1002/aic.14829.

[ref20] Nikolaidis I. K.; Novak N.; Kontogeorgis G. M.; Economou I. G. Rigorous Phase Equilibrium Calculation Methods for Strong Electrolyte Solutions: The Isothermal Flash. Fluid Phase Equilib. 2022, 558, 113441 10.1016/j.fluid.2022.113441.

[ref21] Kontogeorgis G. M.; Schlaikjer A.; Olsen M. D.; Maribo-Mogensen B.; Thomsen K.; von Solms N.; Liang X. A Review of Electrolyte Equations of State with Emphasis on Those Based on Cubic and Cubic-Plus-Association (CPA) Models. Int. J. Thermophys. 2022, 43, 54 10.1007/s10765-022-02976-4.

[ref22] Novak N.; Kontogeorgis G. M.; Castier M.; Economou I. G. Modeling of Gas Solubility in Aqueous Electrolyte Solutions with the eSAFT-VR Mie Equation of State. Ind. Eng. Chem. Res. 2021, 60, 15327–15342. 10.1021/acs.iecr.1c02923. PMC10472441 37663168

[ref23] Jiang H.; Mester Z.; Moultos O. A.; Economou I. G.; Panagiotopoulos A. Z. Thermodynamic and Transport Properties of H_2_O + NaCl from Polarizable Force Fields. J. Chem. Theory Comput. 2015, 11, 3802–3810. 10.1021/acs.jctc.5b00421. 26574461

[ref24] Orozco G. A.; Moultos O. A.; Jiang H.; Economou I. G.; Panagiotopoulos A. Z. Molecular simulation of thermodynamic and transport properties for the H_2_O+NaCl system. J. Chem. Phys. 2014, 141, 234507 10.1063/1.4903928. 25527948

[ref25] Saravi S. H.; Panagiotopoulos A. Z. Activity Coefficients and Solubilities of NaCl in Water–Methanol Solutions from Molecular Dynamics Simulations. J. Phys. Chem. B 2022, 126, 2891–2898. 10.1021/acs.jpcb.2c00813. 35411772

[ref26] Zhang C.; Yue S.; Panagiotopoulos A. Z.; Klein M. L.; Wu X. Dissolving salt is not equivalent to applying a pressure on water. Nat. Commun. 2022, 13, 822 10.1038/s41467-022-28538-8. 35145131 PMC8831556

[ref27] Tham M. J.; Walker R. D. Jr; Gubbins K. E. Diffusion of oxygen and hydrogen in aqueous potassium hydroxide solutions. J. Phys. Chem. 1970, 74, 1747–1751. 10.1021/j100703a015.

[ref28] Tjarks G.; Mergel J.; Stolten D. Hydrogen Science and Engineering: Materials, Processes, Systems and Technology; John Wiley & Sons, Ltd., 2016; Chapter 14, pp 309–330.

[ref29] Manabe A.; Kashiwase M.; Hashimoto T.; Hayashida T.; Kato A.; Hirao K.; Shimomura I.; Nagashima I. Basic study of alkaline water electrolysis. Electrochim. Acta 2013, 100, 249–256. 10.1016/j.electacta.2012.12.105.

[ref30] Bidault F.; Brett D.; Middleton P.; Brandon N. Review of gas diffusion cathodes for alkaline fuel cells. J. Power Sources 2009, 187, 39–48. 10.1016/j.jpowsour.2008.10.106.

[ref31] Tsimpanogiannis I. N.; Maity S.; Celebi A. T.; Moultos O. A. Engineering Model for Predicting the Intradiffusion Coefficients of Hydrogen and Oxygen in Vapor, Liquid, and Supercritical Water based on Molecular Dynamics Simulations. Journal of Chemical & Engineering Data 2021, 66, 3226–3244. 10.1021/acs.jced.1c00300.

[ref32] Chen B.; Ivanov I.; Park J. M.; Parrinello M.; Klein M. L. Solvation Structure and Mobility Mechanism of OH-: A Car- Parrinello Molecular Dynamics Investigation of Alkaline Solutions. J. Phys. Chem. B 2002, 106, 12006–12016. 10.1021/jp026504w.

[ref33] Megyes T.; Bálint S.; Grósz T.; Radnai T.; Bakó I.; Sipos P. The structure of aqueous sodium hydroxide solutions: A combined solution x-ray diffraction and simulation study. J. Chem. Phys. 2008, 128, 044501 10.1063/1.2821956. 18247963

[ref34] Tuckerman M. E.; Chandra A.; Marx D. Structure and dynamics of OH^–^(aq). Acc. Chem. Res. 2006, 39, 151–158. 10.1021/ar040207n. 16489735

[ref35] Guevara-Carrion G.; Nieto-Draghi C.; Vrabec J.; Hasse H. Prediction of Transport Properties by Molecular Simulation: Methanol and Ethanol and Their Mixture. J. Phys. Chem. B 2008, 112, 16664–16674. 10.1021/jp805584d. 19367909

[ref36] Ghaffari A.; Rahbar-Kelishami A. MD simulation and evaluation of the self-diffusion coefficients in aqueous NaCl solutions at different temperatures and concentrations. J. Mol. Liq. 2013, 187, 238–245. 10.1016/j.molliq.2013.08.004.

[ref37] Saravi S. H.; Panagiotopoulos A. Z. Individual Ion Activity Coefficients in Aqueous Electrolytes from Explicit-Water Molecular Dynamics Simulations. J. Phys. Chem. B 2021, 125, 8511–8521. 10.1021/acs.jpcb.1c04019. 34319101

[ref38] Zeron I.; Abascal J.; Vega C. A force field of Li^+^, Na^+^, K^+^, Mg^2+^, Ca^2+^, Cl^–^, and SO_4_^2–^ in aqueous solution based on the TIP4P/2005 water model and scaled charges for the ions. J. Chem. Phys. 2019, 151, 134504 10.1063/1.5121392. 31594349

[ref39] Jiang H.; Moultos O. A.; Economou I. G.; Panagiotopoulos A. Z. Hydrogen-Bonding Polarizable Intermolecular Potential Model for Water. J. Phys. Chem. B 2016, 120, 12358–12370. 10.1021/acs.jpcb.6b08205. 27807969

[ref40] Jiang H.; Moultos O. A.; Economou I. G.; Panagiotopoulos A. Z. Gaussian-Charge Polarizable and Nonpolarizable Models for CO_2_. J. Phys. Chem. B 2016, 120, 984–994. 10.1021/acs.jpcb.5b11701. 26788614

[ref41] Kiss P. T.; Baranyai A. A new polarizable force field for alkali and halide ions. J. Chem. Phys. 2014, 141, 114501 10.1063/1.4895129. 25240358

[ref42] Abascal J. L.; Vega C. A general purpose model for the condensed phases of water: TIP4P/2005. J. Chem. Phys. 2005, 123, 234505 10.1063/1.2121687. 16392929

[ref43] Abascal J. L.; Vega C. Widom line and the liquid–liquid critical point for the TIP4P/2005 water model. J. Chem. Phys. 2010, 133, 234502 10.1063/1.3506860. 21186870

[ref44] Tsimpanogiannis I. N.; Moultos O. A.; Franco L. F. M.; Spera M. B. M.; Erdős M.; Economou I. G. Self-diffusion coefficient of bulk and confined water: a critical review of classical molecular simulation studies. Mol. Simul. 2019, 45, 425–453. 10.1080/08927022.2018.1511903.

[ref45] Blazquez S.; Conde M. M.; Abascal J. L. F.; Vega C. The Madrid-2019 force field for electrolytes in water using TIP4P/2005 and scaled charges: Extension to the ions F^–^, Br^–^, I^–^, Rb^+^, and Cs^+^. J. Chem. Phys. 2022, 156, 044505 10.1063/5.0077716. 35105066

[ref46] Kann Z.; Skinner J. A scaled-ionic-charge simulation model that reproduces enhanced and suppressed water diffusion in aqueous salt solutions. J. Chem. Phys. 2014, 141, 104507 10.1063/1.4894500. 25217937

[ref47] Blazquez S.; Conde M. M.; Vega C. 2023, in preparation.

[ref48] Bonthuis D. J.; Mamatkulov S. I.; Netz R. R. Optimization of classical nonpolarizable force fields for OH^–^ and H_3_O^+^. J. Chem. Phys. 2016, 144, 104503 10.1063/1.4942771. 26979693

[ref49] Hub J. S.; Wolf M. G.; Caleman C.; van Maaren P. J.; Groenhof G.; van der Spoel D. Thermodynamics of hydronium and hydroxide surface solvation. Chemical Science 2014, 5, 1745–1749. 10.1039/c3sc52862f.

[ref50] Pliego J. R.; Riveros J. M. On the Calculation of the Absolute Solvation Free Energy of Ionic Species: Application of the Extrapolation Method to the Hydroxide Ion in Aqueous Solution. J. Phys. Chem. B 2000, 104, 5155–5160. 10.1021/jp000041h.

[ref51] Ufimtsev I. S.; Kalinichev A. G.; Martinez T. J.; Kirkpatrick R. J. A charged ring model for classical OH^–^(aq) simulations. Chem. Phys. Lett. 2007, 442, 128–133. 10.1016/j.cplett.2007.05.042.

[ref52] Botti A.; Bruni F.; Imberti S.; Ricci M. A.; Soper A. K. Ions in water: The microscopic structure of concentrated NaOH solutions. J. Chem. Phys. 2004, 120, 10154–10162. 10.1063/1.1705572. 15268038

[ref53] Imberti S.; Botti A.; Bruni F.; Cappa G.; Ricci M. A.; Soper A. K. Ions in water: The microscopic structure of concentrated hydroxide solutions. J. Chem. Phys. 2005, 122, 194509 10.1063/1.1899147. 16161599

[ref54] Vácha R.; Megyes T.; Bakó I.; Pusztai L.; Jungwirth P. Benchmarking Polarizable Molecular Dynamics Simulations of Aqueous Sodium Hydroxide by Diffraction Measurements. J. Phys. Chem. A 2009, 113, 4022–4027. 10.1021/jp810399p. 19209921

[ref55] Coste A.; Poulesquen A.; Diat O.; Dufrêche J.-F.; Duvail M. Investigation of the Structure of Concentrated NaOH Aqueous Solutions by Combining Molecular Dynamics and Wide-Angle X-ray Scattering. J. Phys. Chem. B 2019, 123, 5121–5130. 10.1021/acs.jpcb.9b00495. 31141363

[ref56] Zapałowski M.; Bartczak W. M. Structural and dynamical properties of concentrated aqueous NaOH solutions: a computer simulation study. Computers & Chemistry 2000, 24, 459–468. 10.1016/S0097-8485(99)00084-4. 10816015

[ref57] Rahbari A.; Hens R.; Ramdin M.; Moultos O. A.; Dubbeldam D.; Vlugt T. J. H. Recent advances in the Continuous Fractional Component Monte Carlo methodology. Mol. Simul. 2021, 47, 804–823. 10.1080/08927022.2020.1828585.

[ref58] Shi W.; Maginn E. J. Continuous Fractional Component Monte Carlo: an adaptive biasing method for open system atomistic simulations. J. Chem. Theory Comput. 2007, 3, 1451–1463. 10.1021/ct7000039. 26633216

[ref59] Shi W.; Maginn E. J. Improvement in molecule exchange efficiency in Gibbs ensemble Monte Carlo: Development and implementation of the Continuous Fractional Component move. J. Comput. Chem. 2008, 29, 2520–2530. 10.1002/jcc.20977. 18478586

[ref60] Moultos O. A.; Tsimpanogiannis I. N.; Panagiotopoulos A. Z.; Economou I. G. Atomistic Molecular Dynamics Simulations of CO_2_ Diffusivity in H_2_O for a Wide Range of Temperatures and Pressures. J. Phys. Chem. B 2014, 118, 5532–5541. 10.1021/jp502380r. 24749622

[ref61] Michalis V. K.; Moultos O. A.; Tsimpanogiannis I. N.; Economou I. G. Molecular dynamics simulations of the diffusion coefficients of light n-alkanes in water over a wide range of temperature and pressure. Fluid Phase Equilib. 2016, 407, 236–242. 10.1016/j.fluid.2015.05.050.

[ref62] Bohn M.; Lustig R.; Fischer J. Description of polyatomic real substances by two-center Lennard-Jones model fluids. Fluid Phase Equilib. 1986, 25, 251–262. 10.1016/0378-3812(86)80001-2.

[ref63] Koster A.; Thol M.; Vrabec J. Molecular models for the hydrogen age: hydrogen, nitrogen, oxygen, argon, and water. Journal of Chemical & Engineering Data 2018, 63, 305–320. 10.1021/acs.jced.7b00706.

[ref64] Marx D.; Nielaba P. Path-integral Monte Carlo techniques for rotational motion in two dimensions: Quenched, annealed, and no-spin quantum-statistical averages. Phys. Rev. A 1992, 45, 8968 10.1103/PhysRevA.45.8968. 9907002

[ref65] Allen M.; Tildesley D.; Tildesley D. Computer Simulation of Liquids, 2nd ed.; Oxford Science Publications; Oxford University Press: New York, 2017.

[ref66] Frenkel D.; Smit B. Understanding Molecular Simulation: From Algorithms to Applications, 2nd ed.; Academic Press: San Diego, CA, 2002.

[ref67] Plimpton S. Fast parallel algorithms for short-range molecular dynamics. J. Comput. Phys. 1995, 117, 1–19. 10.1006/jcph.1995.1039.

[ref68] Verlet L. Computer “Experiments” on Classical Fluids. I. Thermodynamical Properties of Lennard-Jones molecules. Phys. Rev. 1967, 159, 98–103. 10.1103/PhysRev.159.98.

[ref69] Ryckaert J.; Ciccotti G.; Berendsen H. J. Numerical integration of the cartesian equations of motion of a system with constraints: molecular dynamics of n-alkanes. J. Comput. Phys. 1977, 23, 327–341. 10.1016/0021-9991(77)90098-5.

[ref70] Hockney R. W.; Eastwood J. W. Computer Simulation Using Particles; CRC Press: Boca Raton, FL, 1988.

[ref71] Jamali S. H.; Wolff L.; Becker T. M.; De Groen M.; Ramdin M.; Hartkamp R.; Bardow A.; Vlugt T. J. H.; Moultos O. A. OCTP: A tool for on-the-fly calculation of transport properties of fluids with the order-n algorithm in LAMMPS. J. Chem. Inf. Model. 2019, 59, 1290–1294. 10.1021/acs.jcim.8b00939. 30742429

[ref72] Nosé S. A Unified Formulation of the Constant Temperature Molecular Dynamics Methods. J. Chem. Phys. 1984, 81, 511–519. 10.1063/1.447334.

[ref73] Hoover W. G. Canonical Dynamics: Equilibrium Phase-Space Distributions. Phys. Rev. A 1985, 31, 1695 10.1103/PhysRevA.31.1695. 9895674

[ref74] Kamberaj H.; Low R.; Neal M. Time reversible and symplectic integrators for molecular dynamics simulations of rigid molecules. J. Chem. Phys. 2005, 122, 224114 10.1063/1.1906216. 15974658

[ref75] Martínez L.; Andrade R.; Birgin E. G.; Martínez J. M. PACKMOL: A package for building initial configurations for molecular dynamics simulations. J. Comput. Chem. 2009, 30, 2157–2164. 10.1002/jcc.21224. 19229944

[ref76] Yeh I.; Hummer G. System-size dependence of diffusion coefficients and viscosities from molecular dynamics simulations with periodic boundary conditions. J. Phys. Chem. B 2004, 108, 15873–15879. 10.1021/jp0477147.

[ref77] Dünweg B.; Kremer K. Molecular dynamics simulation of a polymer chain in solution. J. Chem. Phys. 1993, 99, 6983–6997. 10.1063/1.465445.

[ref78] Celebi A. T.; Jamali S. H.; Bardow A.; Vlugt T. J. H.; Moultos O. A. Finite-size effects of diffusion coefficients computed from molecular dynamics: a review of what we have learned so far. Mol. Simul. 2021, 47, 831–845. 10.1080/08927022.2020.1810685.

[ref79] Jamali S. H.; Bardow A.; Vlugt T. J. H.; Moultos O. A. Generalized form for finite-size corrections in mutual diffusion coefficients of multicomponent mixtures obtained from equilibrium molecular dynamics simulation. J. Chem. Theory Comput. 2020, 16, 3799–3806. 10.1021/acs.jctc.0c00268. 32338889 PMC7288667

[ref80] Jamali S. H.; Wolff L.; Becker T. M.; Bardow A.; Vlugt T. J. H.; Moultos O. A. Finite-Size Effects of Binary Mutual Diffusion Coefficients from Molecular Dynamics. J. Chem. Theory Comput. 2018, 14, 2667–2677. 10.1021/acs.jctc.8b00170. 29664633 PMC5943679

[ref81] Jamali S. H.; Hartkamp R.; Bardas C.; Söhl J.; Vlugt T. J. H.; Moultos O. A. Shear Viscosity Computed from the Finite-Size Effects of Self-Diffusivity in Equilibrium Molecular Dynamics. J. Chem. Theory Comput. 2018, 14, 5959–5968. 10.1021/acs.jctc.8b00625. 30296092 PMC6236468

[ref82] Moultos O. A.; Zhang Y.; Tsimpanogiannis I. N.; Economou I. G.; Maginn E. J. System-size corrections for self-diffusion coefficients calculated from molecular dynamics simulations: The case of CO_2_, n-alkanes, and poly(ethylene glycol) dimethyl ethers. J. Chem. Phys. 2016, 145, 074109 10.1063/1.4960776. 27544089

[ref83] Salehi H. S.; Hens R.; Moultos O. A.; Vlugt T. J. H. Computation of gas solubilities in choline chloride urea and choline chloride ethylene glycol deep eutectic solvents using Monte Carlo simulations. J. Mol. Liq. 2020, 316, 113729 10.1016/j.molliq.2020.113729.

[ref84] Hens R.; Rahbari A.; Caro-Ortiz S.; Dawass N.; Erdős M.; Poursaeidesfahani A.; Salehi H. S.; Celebi A. T.; Ramdin M.; Moultos O. A.; Dubbeldam D.; Vlugt T. J. H. Brick-CFCMC: Open Source Software for Monte Carlo Simulations of Phase and Reaction Equilibria Using the Continuous Fractional Component Method. J. Chem. Inf. Model. 2020, 60, 2678–2682. 10.1021/acs.jcim.0c00334. 32275829 PMC7312392

[ref85] Polat H. M.; Salehi H. S.; Hens R.; Wasik D. O.; Rahbari A.; de Meyer F.; Houriez C.; Coquelet C.; Calero S.; Dubbeldam D.; Moultos O. A.; Vlugt T. J. H. New Features of the Open Source Monte Carlo Software Brick-CFCMC: Thermodynamic Integration and Hybrid Trial Moves. J. Chem. Inf. Model. 2021, 61, 3752–3757. 10.1021/acs.jcim.1c00652. 34383501 PMC8385706

[ref86] Rahbari A.; Brenkman J.; Hens R.; Ramdin M.; Van Den Broeke L. J.; Schoon R.; Henkes R.; Moultos O. A.; Vlugt T. J. H. Solubility of water in hydrogen at high pressures: a molecular simulation study. Journal of Chemical & Engineering Data 2019, 64, 4103–4115. 10.1021/acs.jced.9b00513.

[ref87] Poursaeidesfahani A.; Hens R.; Rahbari A.; Ramdin M.; Dubbeldam D.; Vlugt T. J. H. Efficient application of Continuous Fractional Component Monte Carlo in the reaction ensemble. J. Chem. Theory Comput. 2017, 13, 4452–4466. 10.1021/acs.jctc.7b00092. 28737933 PMC5597954

[ref88] Rahbari A.; Hens R.; Dubbeldam D.; Vlugt T. J. H. Improving the accuracy of computing chemical potentials in CFCMC simulations. Mol. Phys. 2019, 117, 3493–3508. 10.1080/00268976.2019.1631497.

[ref89] Rahbari A.; Hens R.; Jamali S.; Ramdin M.; Dubbeldam D.; Vlugt T. J. H. Effect of truncating electrostatic interactions on predicting thermodynamic properties of water–methanol systems. Mol. Simul. 2019, 45, 336–350. 10.1080/08927022.2018.1547824.

[ref90] Wang F.; Landau D. P. Efficient multiple-range random walk algorithm to calculate the density of states. Phys. Rev. Lett. 2001, 86, 2050 10.1103/PhysRevLett.86.2050. 11289852

[ref91] Poulain P.; Calvo F.; Antoine R.; Broyer M.; Dugourd P. Performances of Wang-Landau algorithms for continuous systems. Phys. Rev. E 2006, 73, 056704 10.1103/PhysRevE.73.056704. 16803071

[ref92] Heidar-Zadeh F.; Ayers P. W.; Verstraelen T.; Vinogradov I.; Vöhringer-Martinez E.; Bultinck P. Information-Theoretic Approaches to Atoms-in-Molecules: Hirshfeld Family of Partitioning Schemes. J. Phys. Chem. A 2018, 122, 4219–4245. 10.1021/acs.jpca.7b08966. 29148815

[ref93] Gilliam R.; Graydon J.; Kirk D.; Thorpe S. A review of specific conductivities of potassium hydroxide solutions for various concentrations and temperatures. Int. J. Hydrogen Energy 2007, 32, 359–364. 10.1016/j.ijhydene.2006.10.062.

[ref94] Olsson J.; Jernqvist Å.; Aly G. Thermophysical properties of aqueous NaOH-H_2_O solutions at high concentrations. Int. J. Thermophys. 1997, 18, 779–793. 10.1007/BF02575133.

[ref95] Guo Y.; Xu H.; Guo F.; Zheng S.; Zhang Y. Density and viscosity of aqueous solution of K_2_CrO_4_/KOH mixed electrolytes. Transactions of Nonferrous Metals Society of China 2010, 20, s32–s36. 10.1016/S1003-6326(10)60007-6.

[ref96] Marcus Y. Ionic radii in aqueous solutions. Chem. Rev. 1988, 88, 1475–1498. 10.1021/cr00090a003.

[ref97] Yuan-Hui L.; Gregory S. Diffusion of ions in sea water and in deep-sea sediments. Geochim. Cosmochim. Acta 1974, 38, 703–714. 10.1016/0016-7037(74)90145-8.

[ref98] Tuckerman M.; Laasonen K.; Sprik M.; Parrinello M. Ab Initio Molecular Dynamics Simulation of the Solvation and Transport of H_3_O^+^ and OH^–^ Ions in Water. J. Phys. Chem. 1995, 99, 5749–5752. 10.1021/j100016a003.

[ref99] Walker R. D. Jr. Study of Gas Solubilities and Transport Properties in Fuel Cell Electrolytes; Technical Report; Florida University, Gainesville, FL, 1971.

[ref100] Setschenow J. Über die konstitution der salzlö sungen auf grund ihres verhaltens zu kohlensäure. Z. Phys. Chem. 1889, 4, 117–125. 10.1515/zpch-1889-0109.

[ref101] Weisenberger S.; Schumpe A. Estimation of gas solubilities in salt solutions at temperatures from 273 to 363 K. AIChE J. 1996, 42, 298–300. 10.1002/aic.690420130.

[ref102] Davis R.; Horvath G.; Tobias C. The solubility and diffusion coefficient of oxygen in potassium hydroxide solutions. Electrochim. Acta 1967, 12, 287–297. 10.1016/0013-4686(67)80007-0.

[ref103] Shoor S.; Walker R. D. Jr; Gubbins K. Salting out of nonpolar gases in aqueous potassium hydroxide solutions. J. Phys. Chem. 1969, 73, 312–317. 10.1021/j100722a006.

[ref104] Ruetschi P.; Amlie R. Solubility of hydrogen in potassium hydroxide and sulfuric acid. Salting-out and hydration. J. Phys. Chem. 1966, 70, 718–723. 10.1021/j100875a018.

