# Supplementary material for: A New Force Field for OH– for Computing Thermodynamic and Transport Properties of H2 and O2 in Aqueous NaOH and KOH Solutions
Source: J Phys Chem B. 2022 Nov 3;126(45):9376–87. doi: 10.1021/acs.jpcb.2c06381 (PMC9677430; doi:10.1021/acs.jpcb.2c06381)
Supplement: Supplementary file 1 — jp2c06381_si_001.pdf [file jp2c06381_si_001.pdf]

Supporting Information: A New Force Field for OH^- for Computing Thermodynamic and Transport Properties of H_2 and O_2 in Aqueous NaOH and KOH Solutions

Parsa Habibi,[†,‡] Ahmadreza Rahbari,[†] Samuel Blazquez,[¶] Carlos Vega,[¶] Poulumi Dey,[‡] Thijs J. H. Vlugt,[†] and Othonas A. Moultos[*,†]

†*Engineering Thermodynamics, Process & Energy Department, Faculty of Mechanical, Maritime and Materials Engineering, Delft University of Technology, Leeghwaterstraat 39, 2628 CB Delft, The Netherlands*

‡*Department of Materials Science and Engineering, Faculty of Mechanical, Maritime and Materials Engineering, Delft University of Technology, Mekelweg 2, 2628 CD Delft, The Netherlands*

¶*Depto. Química Física, Fac. Ciencias Químicas, Universidad Complutense de Madrid, 28040 Madrid, Spain*

E-mail: O.moultos@tudelft.nl

Comparison of the Marx[1] and Vrabec[2] H_2 force fields for the prediction of H_2 self-diffusivities (finite-size corrected[3]) and solubilities in pure TIP4P/2005 water (Figure S1); Influence of OH^- ion size on densities, and viscosities at 1 bar and 298 K (Figure S2); Radial Distribution Functions (RDFs) of cation-anion, cation-anion, and anion-anion of NaOH, and KOH solutions at 1 bar and 298 K (Figure S3); Variation of densities and dynamic viscosities for a pressure range of 1-100 bar at 298 K for different concentrations of aqueous NaOH and KOH (Figure S4); Engineering fits for experimental and simulation data for diffusivities of H_2 and O_2 in aqueous NaOH and KOH solutions (Figure S5); Solubilities of H_2 and O_2 for a pressure range of 1-100 bar at 298 K in pure water calculated using Monte Carlo simulations (Figure S6); Engineering fits for experimental and simulation data for solubilities of H_2 and O_2 in aqueous NaOH and KOH solutions (Figure S7); Force field details for TIP4P/2005 water[4] (Table S1), Vrabec[2] H_2 (Table S2), Marx[1] H_2 (Table S2), and Bohn[5] O_2 (Table S3);[6] Number of Na^+ (Table S4) and K^+ (Table S5) ions used for Molecular Dynamics and Monte Carlo simulations and the respective electrolyte weight percentages (wt%), molalities, and molarities (at 298 K and 1 bar); Raw data for densities, viscosities, and self-diffusivities of H_2 and O_2 at various temperatures and concentrations of aqueous KOH at 1 bar (Table S6) and 100 bar (Table S7); Raw data for densities, viscosities, and self-diffusivities of H_2 and O_2 at various temperatures and concentrations of aqueous NaOH at 1 bar (Table S8), and 100 bar (Table S9); Raw data for excess chemical potentials, Henry coefficients, and solubilities of H_2 and O_2 at various temperatures and concentrations of aqueous KOH (Table S10) and NaOH (Table S11) at 1 bar;

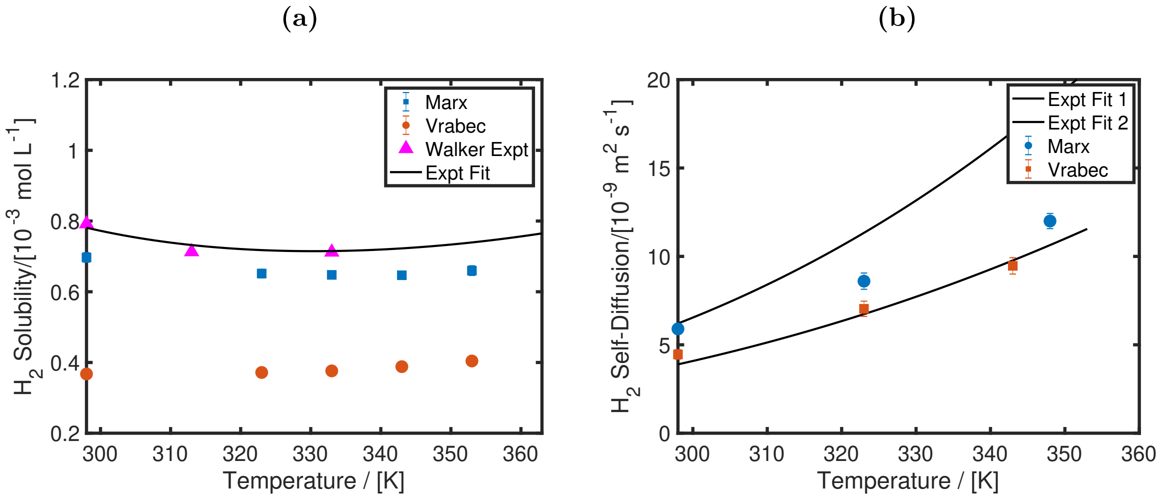

Figure S1: Computed H_2 (a) solubilities and (b) self-diffusivities as a function of temperature in pure water (TIP4P/2005[4]) at 1 bar. The H_2 solubilities are calculated using the single site Vrabec force field[2] and the three site Marx force field.[1] The solubilities are computed based on Henry coefficients computed using Continuous Fractional Component (CFC)[7–11] Monte Carlo simulations using Equations 3-5 of the main text. The experimental H_2 solubilities provided by Walker et al.[12] (Triangles), and the experimental fit by Young et al.[13] (shown as a line) are also plotted in (a). The experimental fit of Young et al.[13] provides the solubilities in units of mole fraction. This unit is converted to mol/L using the Olsson fit[14] for densities at different temperatures. Tsimpanogiannis et al.[15] have found that existing experimental data sets for diffusivities of H_2 in pure water at various temperatures at 1 bar fall between two distinct correlations. These correlations are referred to as Exp Fit 1 (upper limit) and Exp Fit 2 (lower limit), and are shown as lines. The H_2 self-diffusivities in water using the Marx force field[1] are computed by Tsimpanogiannis et al.[16] The Marx force field[1] provides a better prediction of the H_2 solubilities and is used further for solubility predictions in aqueous NaOH and KOH solutions. For H_2 self-diffusivities, both force fields provide similar results. The Vrabec force field[2] is used for self-diffusion predictions as it allows the use of the SHAKE algorithm[17] in LAMMPS.[18] For the Marx force field[1] (linear model with three sites), the SHAKE algorithm in LAMMPS does not work.[17]

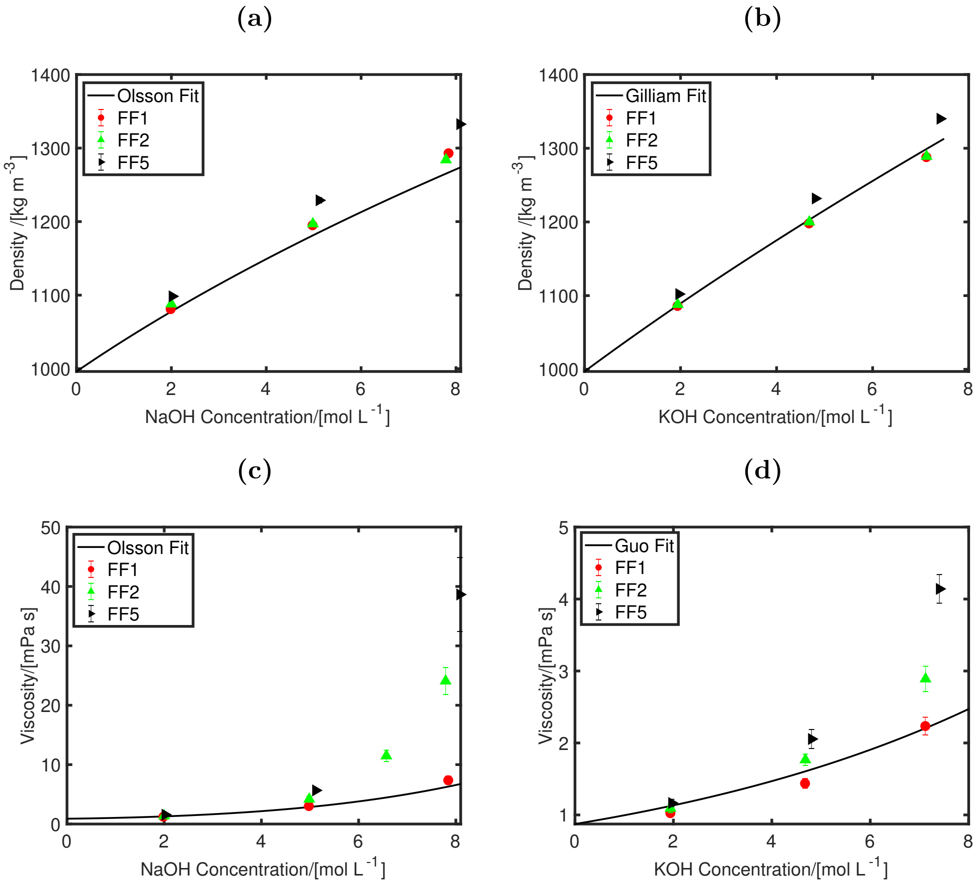

Figure S2: Computed densities ((a), (b)), and viscosities ((c), (d)) of aqueous NaOH ((a), (c)), and KOH ((b), (d)) solutions as functions of electrolyte concentrations at 298 K, and 1 bar. The TIP4P/2005 water model,[4] and the Madrid-Transport Na^+ and K^+ models[19] are used for the MD simulations. Three different OH^- models are shown in (a)-(d). FF1 has a total charge of -0.75 [e], with a σ_{OO} (Lennard-Jones sigma parameter of the O atom in OH^-) of 3.65 Å. FF2 has a total charge of -0.85 [e], with a σ_{OO} of 3.85 Å, and FF5 has a total charge of -0.85 [e], with a σ_{OO} of 3.65 Å. All three models have a ϵ_{OO}/k_B (Lennard-Jones epsilon parameter of the O atom in OH^-) of 30.19 K, a ϵ_{HH}/k_B (Lennard-Jones epsilon parameter of the H atom in OH^-) of 22.13 K, and a σ_{HH} (Lennard-Jones sigma parameter of the H atom in OH^-) of 1.443 Å. All the details of the FF1 and FF2 model are shown in Table 2 of the main manuscript. FF5 has the same q_O (charge of O atom in OH^-) and q_H (charge of H atom in OH^-) as the FF2 model. Changing the total charge (q_{OH}) from -0.75 to -0.85, while keeping the σ_{OO} constant (comparison between FF1, and FF5) leads to a slight increase in densities, and a significant increase in the computed viscosities. Reducing the σ_{OO} constant from 3.85 Å, to 3.65 Å at constant q_{OH} (and partial charges), also leads to an increase in the computed densities, and shear viscosities. Decreasing σ_{OO} (i.e., shifting the first hydration shell closer towards the O-atom), and changing q_{OH} from -0.75 to -0.85, lowers the total volume of the system, and increases the interaction energy between the OH^- and the molecules in its first hydration shell. The experimental correlations of Gilliam et al.[20] (densities), and Guo et al.[21] (dynamic viscosities) are used for aqueous KOH solutions at 298 K and 1 bar and are plotted as lines. For aqueous NaOH solutions, the experimental correlations of Olsson et al.[14] are used for densities and dynamic viscosities.

(a)

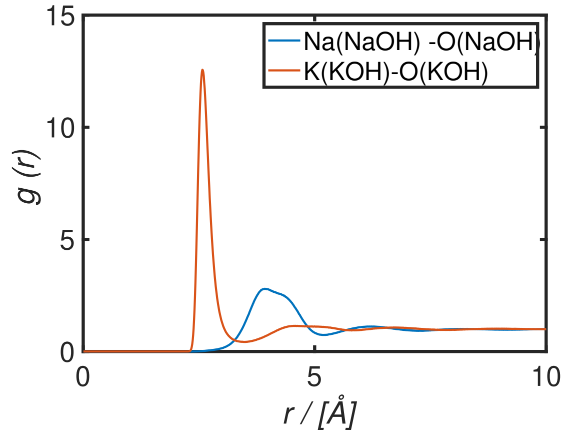

(b)

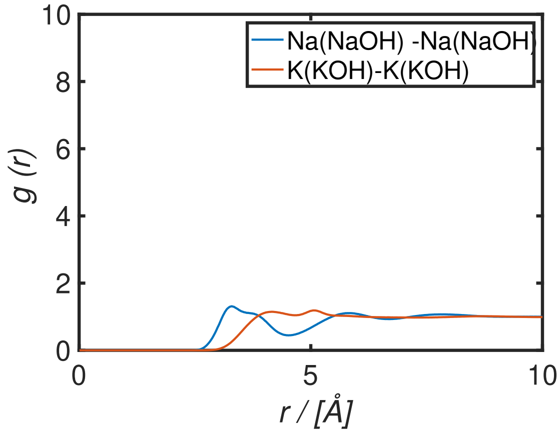

(c)

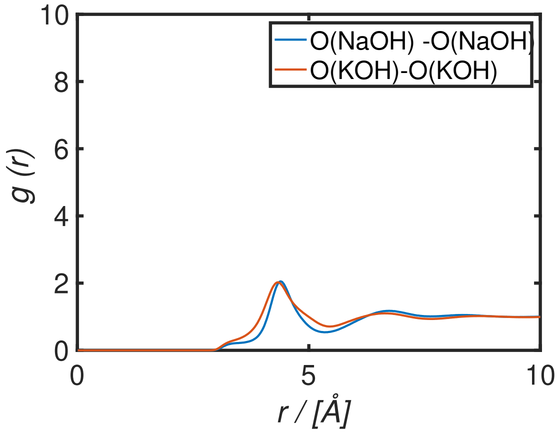

Figure S3: Computed radial distribution functions ($g(r)$) for (a) Na^+(NaOH)−O(NaOH) and K^+(KOH)−O(KOH), and (b) Na^+(NaOH)-Na^+(NaOH) and K^+(KOH)-K^+(KOH), and (c) O(NaOH)-O(NaOH) and O(KOH)-O(KOH) as functions of radial distance r (Å), at 298 K, 1 bar, and a concentration of 5 mol/kg (corresponding to a molarity of 4.98 mol/L for NaOH, and 4.68 mol/L KOH). The FF1 OH^- model (as shown in Table 2 of the main manuscript), in combination with the TIP4P/2005 water model,[4] and the Madrid-Transport Na^+, and K^+ models[19] are used for the MD simulations.

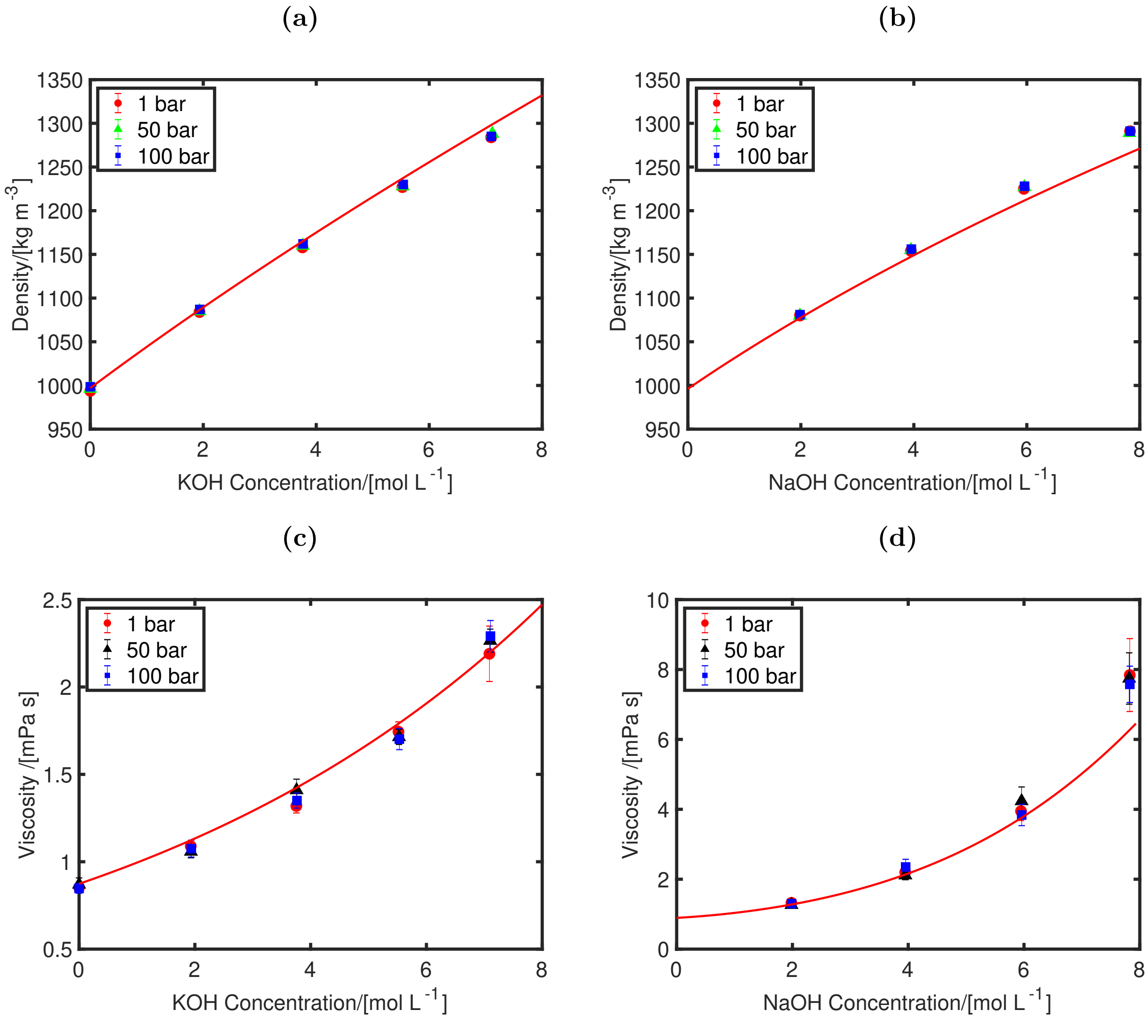

Figure S4: Computed densities (a)-(b) and dynamic viscosities (c)-(d) of alkaline solutions as a function of KOH ((a),(c)) and NaOH ((b),(d)) concentrations at 298 K. Three different pressures are considered, i.e. 1, 50, and 100 bar. The experimental correlations of Gilliam et al.[20] (densities), and Guo et al.[21] (dynamic viscosities) are used for aqueous KOH solutions at 298 K and 1 bar and plotted as lines. For aqueous NaOH solutions, the experimental correlations of Olsson et al.[14] are used for densities and dynamic viscosities.

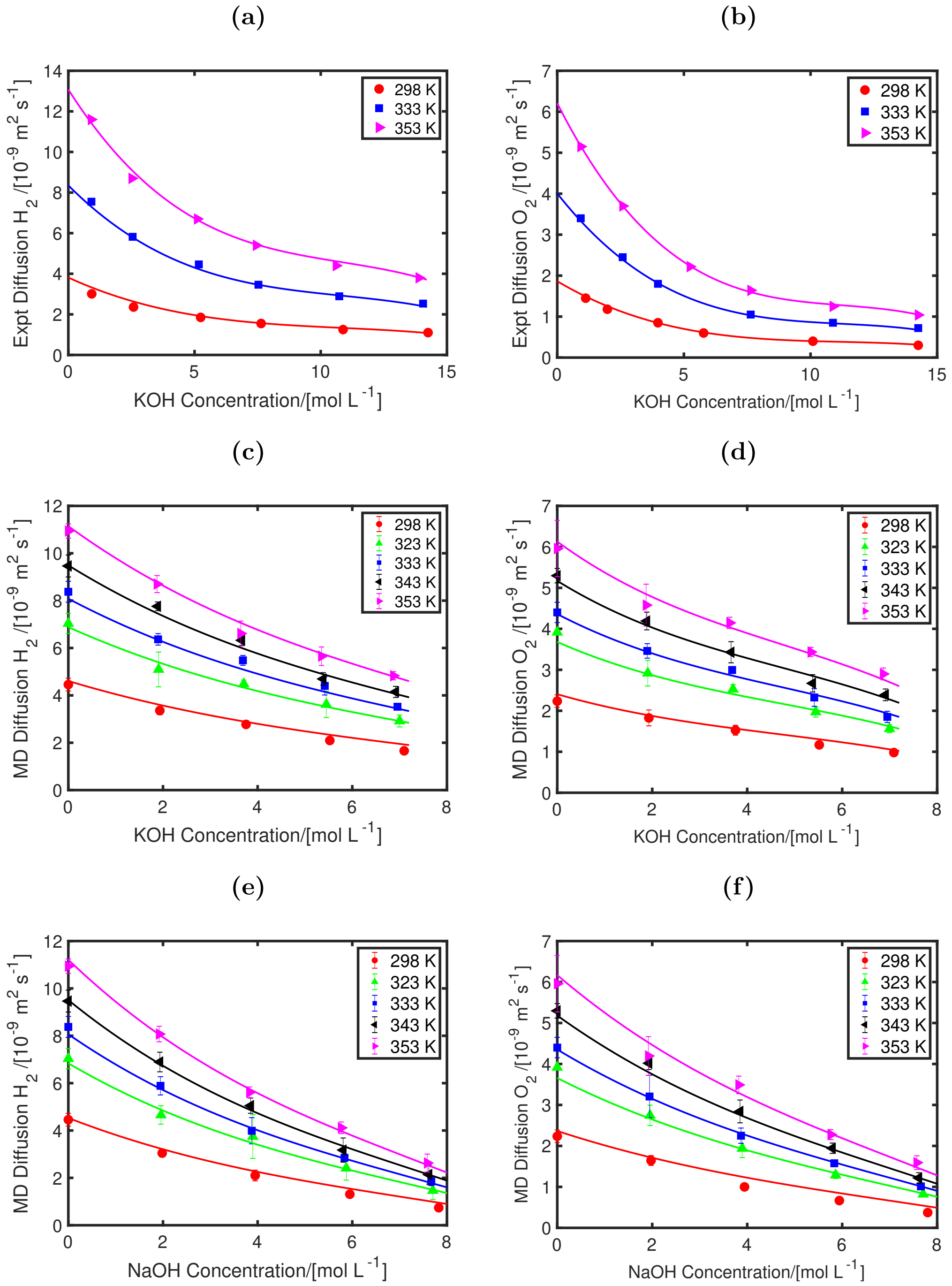

Figure S5: Fitting of computed H_2 and O_2 self-diffusivities as functions of the electrolyte (KOH and NaOH) concentrations and temperatures at 1 bar. All self-diffusivities are system-size corrected using the Yeh-Hummer equation.[3,22–24] Equation 7 of the main text is used for fitting the experimental data (a)-(b) of Tham et al.[12,25] and the simulation results (c)-(f). These fits are shown as lines. Tham et al.[12,25] report H_2 and O_2 solubilities at different temperatures as a function of concentrations in units of KOH weight percent (wt%). This unit is converted to mol/L using the density correlation provided by Gilliam et al.[20] The engineering equations for the self-diffusivities (as discussed in the main text), as shown by the lines, provide excellent fits for both the experiments and simulation results.

(a)

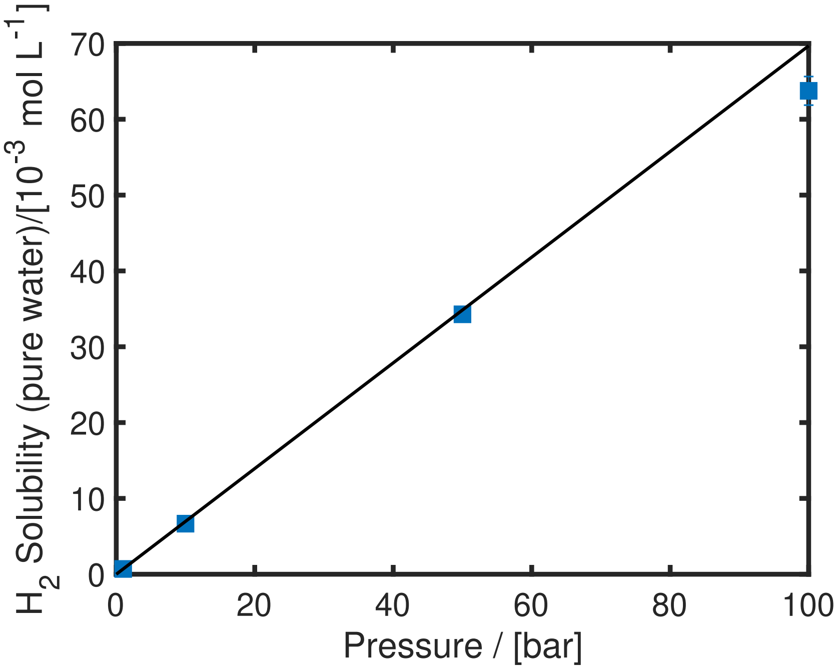

(b)

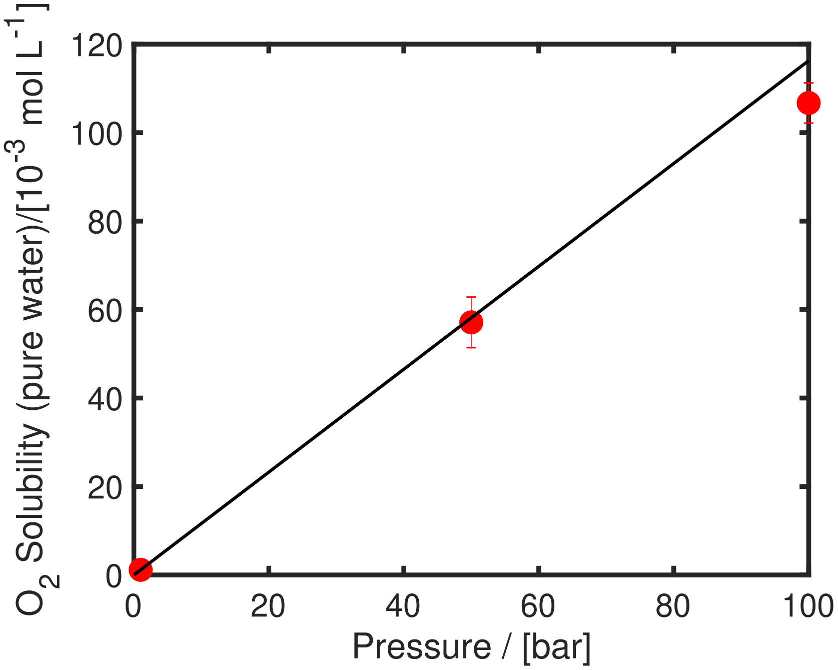

Figure S6: Computed solubilities of the (a) H_2 (Marx force field[1]), and (b) O_2(Bohn force field[5]) in water (TIP4P/2005[4]) as functions of H_2 and O_2 partial pressure at 298 K. The solubilities are computed based on Henry coefficients computed using Continuous Fractional Component (CFC)[7–11] Monte Carlo simulations using Equations 3-5 of the main text. Up to ca. 100 bar, the solubilities can be described using Henry's law.

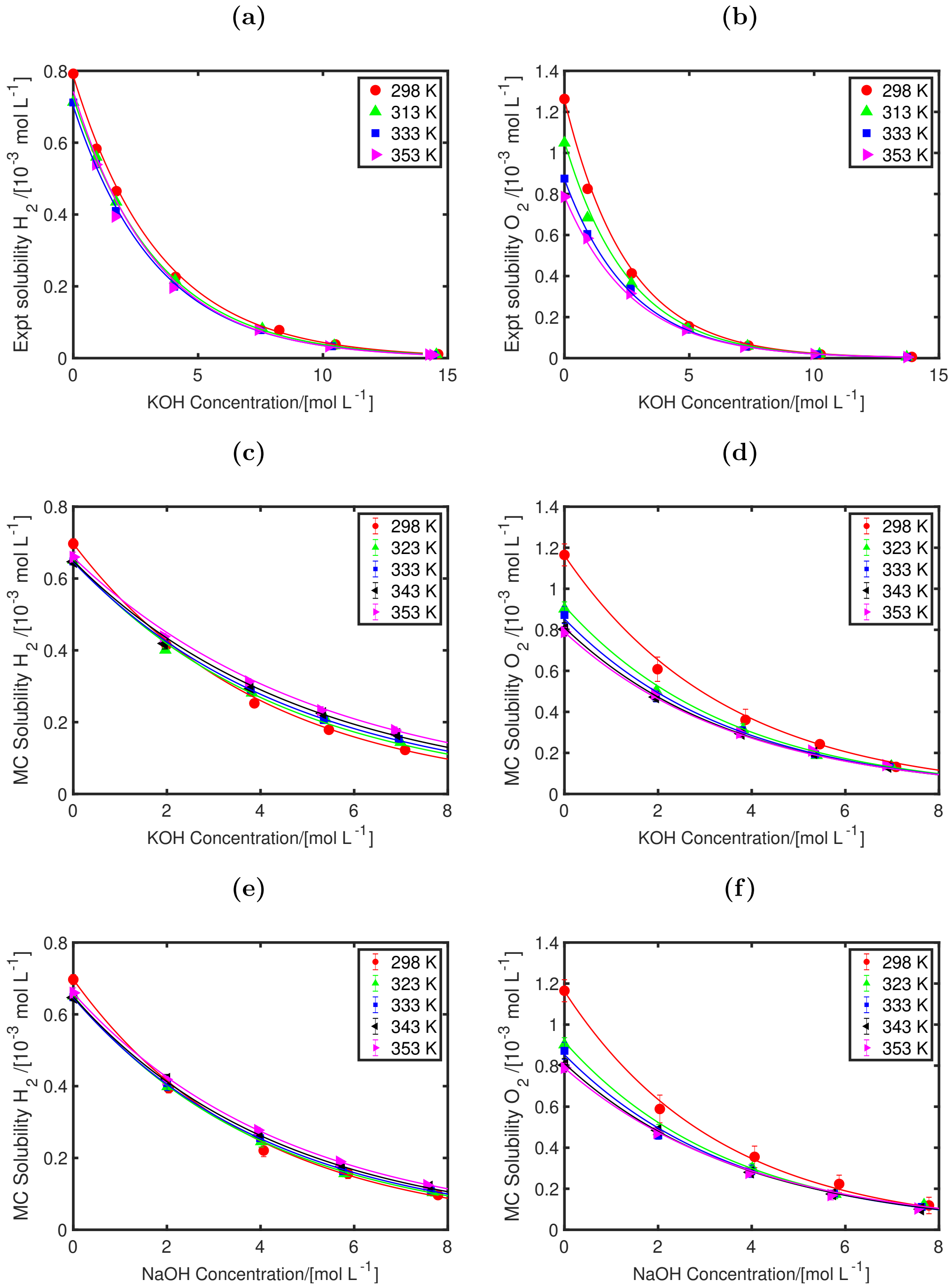

Figure S7: Fitting of computed H_2, and O_2 solubilities as functions of the electrolyte (NaOH and KOH) concentrations and temperatures at H_2 and O_2 partial pressures of 1 bar. Equations 8-9 of the main text are used for fitting the experimental data (a)-(b) of Walker et al.[12] and the simulation results (c)-(f). These fits are shown as lines. Walker et al.[12] show H_2 and O_2 solubilities at different temperatures as a function of concentrations in units of KOH weight percent (wt%). This unit is converted to mol/L using the density correlation provided by Gilliam et al.[20] The engineering equations for the solubilities (as discussed in the main manuscript), indicated by the lines, provide excellent fits for both the experiments and simulation results.

Table S1: Force field parameters for the TIP4P/2005[4] rigid water model. ϵ and σ are the Lennard-Jones parameters and the values of q are the atomic partial charges. $l_{\mathrm{O-H}}$ is the $\mathrm{O-H}$ bond length and H-$\widehat{\mathrm{O}}$-H is the H_2O bond angle.

H-$\widehat{\mathrm{O}}$-H / [°]	104.52
$l_{\mathrm{O-H}}$ / [Å]	0.9572
σ_{HH} / [Å]	0
σ_{OO} / [Å]	3.1589
$\epsilon_{\mathrm{HH}}/k_B$ / [K]	0
$\epsilon_{\mathrm{OO}}/k_B$ / [K]	93.2
q_{H} / [e]	0.5564
q_{O} / [e]	-1.1128

Table S2: Force field parameters for the hydrogen (H_2) models used. ϵ and σ are the Lennard-Jones parameters and the values of q are the atomic partial charges. For the three-site Marx model,[1] the bond length is fixed to 0.74Å. L represents the dummy site in the Marx model, which is placed at the center of the two H atoms.

	Vrabec[2]	Marx[1]
$\epsilon_{\mathrm{HH}}/k_B$ / [K]	25.84	-
σ_{HH}/ [Å]	3.0366	-
q_{L}/ [e]	-	-0.936
q_{H}/ [e]	-	0.468
$\epsilon_{\mathrm{LL}}/k_B$ / [K]	-	36.7
σ_{LL}/ [Å]	-	2.958

Table S3: Force field parameters for the two-site Bohn model for O_2.[5] ϵ and σ are the Lennard-Jones parameters and q is the atomic partial charge. The $\mathrm{O-O}$ bond length ($l_{\mathrm{O-O}}$) is fixed at 0.706 Å.

$l_{\mathrm{O-O}}$ / [Å]	0.9572
σ_{OO} / [Å]	3.2104
$\epsilon_{\mathrm{OO}}/k_B$ / [K]	37.99
q_{O} / [e]	0

Table S4: Number of Na^+ ions (which is equal to the number of OH^- ions) used for Molecular Dynamics (MD) and Monte Carlo simulations (MC) and the corresponding molalities (mol/kg), electrolyte weight percentages (wt%), and molarities (mol/L, at 298 K and 1 bar). In MD, the simulation box contains 700 water molecules and in MC there are 300 water molecules. The complete raw data (with molarities, densities, and viscosities) at different temperatures is shown in Tables S8 (1 bar) and S9 (100 bar).

	No. Na^+	Molality (mol/kg water)	Electrolyte wt%	Molarity (mol/L)
MD	25	1.98	0.074	1.98
	50	3.97	0.137	3.94
	76	6.03	0.194	5.94
	101	8.02	0.243	7.80
MC	11	2.04	0.075	2.04
	22	4.07	0.140	4.07
	32	5.93	0.192	5.87
	43	7.96	0.242	7.79

Table S5: Number of K^+ ions (which is equal to the number of OH^- ions) used for Molecular Dynamics (MD) and Monte Carlo simulations (MC) and the corresponding molalities (mol/kg), electrolyte weight percentages (wt%), and molarities (mol/L, at 298 K and 1 bar). In MD the simulation box contains 700 water molecules and in MC there are 300 water molecules. The complete raw data (with molarities, densities, and viscosities) at different temperatures is shown in Tables S6 (1 bar) and S7 (100 bar).

	No. K^+	Molality (mol/kg water)	Electrolyte wt%	Molarity (mol/L)
MD	25	1.98	0.100	1.93
	50	3.97	0.182	3.75
	76	6.03	0.253	5.52
	101	8.02	0.310	7.09
MC	11	2.04	0.103	1.99
	22	4.07	0.186	3.87
	32	5.93	0.250	5.46
	43	7.96	0.309	7.09

Table S6: Densities (ρ in units of kg/m^3), dynamic viscosities (η in units of m Pa s), self-diffusivities of hydrogen (D_{s,H_2}, in units of 10^{-9}m^2/s) and oxygen (D_{s,H_2}, in units of 10^{-9}m^2/s) at different temperatures (T, in units of K), and concentrations of KOH at 1 bar. The molalities (m, in units of mol/kg) and molarities (C, in units of mol/L) are shown. The standard deviations (σ) are listed for each respective quantity (in the same unit). The TIP4P/2005 water model,[4] the Vrabec H_2 model,[2] the Bohn O_2 model,[5] the Madrid-Transport model[19] (for K^+), and the newly proposed OH^- model (see the main text) are used for producing these results.

T	m	C	ρ	σ_ρ	η	σ_η	D_{s,H_2}	$\sigma_{D_{s,H_2}}$	D_{s,O_2}	$\sigma_{D_{s,O_2}}$
298.00	0.00	0.00	997.20	1.97	0.85	0.03	4.46	0.27	2.24	0.15
298.00	1.98	1.93	1086.00	0.77	1.09	0.02	3.36	0.18	1.83	0.19
298.00	3.97	3.75	1162.00	1.02	1.32	0.04	2.77	0.15	1.53	0.12
298.00	6.03	5.52	1230.00	0.88	1.74	0.06	2.09	0.15	1.17	0.09
298.00	8.02	7.09	1287.00	0.77	2.19	0.16	1.66	0.12	0.98	0.08
323.00	0.00	0.00	986.90	0.94	0.53	0.03	7.04	0.43	3.92	0.05
323.00	1.98	1.91	1073.00	1.50	0.68	0.01	5.10	0.73	2.92	0.31
323.00	3.97	3.70	1146.00	1.24	0.89	0.05	4.48	0.16	2.53	0.11
323.00	6.03	5.44	1213.00	1.25	1.11	0.03	3.62	0.56	1.97	0.13
323.00	8.02	7.00	1271.00	1.44	1.45	0.12	2.92	0.25	1.56	0.10
333.00	0.00	0.00	982.80	0.73	0.47	0.02	8.38	0.44	4.40	0.25
333.00	1.98	1.90	1067.00	0.61	0.59	0.02	6.37	0.24	3.46	0.18
333.00	3.97	3.68	1140.00	1.00	0.75	0.01	5.48	0.21	3.00	0.08
333.00	6.03	5.41	1206.00	1.16	0.95	0.02	4.40	0.38	2.33	0.22
333.00	8.02	6.95	1262.00	0.84	1.16	0.07	3.52	0.13	1.85	0.14
343.00	0.00	0.00	975.10	0.33	0.41	0.02	9.46	0.46	5.30	0.18
343.00	1.98	1.89	1062.00	0.35	0.51	0.01	7.76	0.18	4.19	0.22
343.00	3.97	3.66	1133.00	0.49	0.64	0.01	6.33	0.22	3.43	0.26
343.00	6.03	5.38	1198.00	1.29	0.86	0.02	4.69	0.27	2.67	0.21
343.00	8.02	6.91	1255.00	0.99	1.01	0.02	4.15	0.23	2.39	0.14
353.00	0.00	0.00	970.30	0.77	0.35	0.01	10.94	0.30	5.96	0.69
353.00	1.98	1.87	1054.00	0.66	0.45	0.01	8.70	0.36	4.58	0.51
353.00	3.97	3.63	1125.00	0.63	0.57	0.02	6.61	0.52	4.15	0.14
353.00	6.03	5.34	1190.00	0.82	0.74	0.01	5.65	0.39	3.43	0.11
353.00	8.02	6.86	1246.00	1.38	0.90	0.04	4.83	0.17	2.90	0.15

Table S7: Densities (ρ in units of kg/m^3), dynamic viscosities (η in units of m Pa s), self-diffusivities of hydrogen (D_{s,H_2}, in units of 10^{-9}m^2/s) and oxygen (D_{s,H_2}, in units of 10^{-9}m^2/s) at different temperatures (T, in units of K), and concentrations of KOH at 100 bar. The molalities (m, in units of mol/kg) and molarities (C, in units of mol/L) are shown. The standard deviations (σ) are listed for each respective quantity (in the same unit). The TIP4P/2005 water model,[4] the Vrabec H_2 model,[2] the Bohn O_2 model,[5] the Madrid-Transport model[19] (for K^+), and the newly proposed OH^- model (see the main text) are used for producing these results.

T	m	C	ρ	σ_ρ	η	σ_η	D_{s,H_2}	$\sigma_{D_{s,H_2}}$	D_{s,O_2}	$\sigma_{D_{s,O_2}}$
298.00	0.00	0.00	1002.00	0.57	0.85	0.03	4.24	0.30	2.19	0.15
298.00	1.98	1.94	1091.00	0.55	1.08	0.05	3.36	0.24	1.79	0.11
298.00	3.97	3.76	1165.00	1.07	1.35	0.04	2.63	0.29	1.30	0.20
298.00	6.03	5.53	1233.00	1.15	1.70	0.06	2.11	0.13	1.21	0.06
298.00	8.02	7.11	1290.00	1.97	2.29	0.09	1.54	0.04	0.91	0.07
323.00	0.00	0.00	992.10	0.59	0.55	0.02	6.81	0.30	3.72	0.19
323.00	1.98	1.91	1077.00	0.61	0.68	0.02	5.68	0.37	3.00	0.17
323.00	3.97	3.71	1150.00	0.64	0.87	0.03	4.29	0.17	2.42	0.17
323.00	6.03	5.46	1217.00	0.96	1.11	0.06	3.48	0.21	2.05	0.13
323.00	8.02	7.00	1271.00	0.74	1.38	0.02	2.95	0.35	1.47	0.27
333.00	0.00	0.00	987.20	0.75	0.46	0.02	7.50	0.36	4.53	0.14
333.00	1.98	1.90	1071.00	1.73	0.60	0.03	6.34	0.27	3.55	0.33
333.00	3.97	3.69	1144.00	0.84	0.78	0.01	5.24	0.26	2.95	0.21
333.00	6.03	5.43	1210.00	1.24	0.97	0.01	4.22	0.34	2.40	0.14
333.00	8.02	6.97	1265.00	1.18	1.20	0.01	3.21	0.38	2.02	0.13
343.00	0.00	0.00	980.60	1.35	0.39	0.01	9.75	0.60	5.13	0.50
343.00	1.98	1.89	1065.00	0.81	0.54	0.03	7.13	0.46	4.19	0.12
343.00	3.97	3.67	1137.00	1.54	0.66	0.03	5.85	0.18	3.37	0.35
343.00	6.03	5.39	1202.00	0.65	0.84	0.03	4.65	0.51	2.77	0.19
343.00	8.02	6.92	1257.00	1.67	1.06	0.03	4.11	0.16	2.31	0.11
353.00	0.00	0.00	974.30	0.87	0.36	0.01	10.54	0.18	6.27	0.36
353.00	1.98	1.88	1058.00	0.99	0.46	0.01	7.98	0.68	4.85	0.25
353.00	3.97	3.65	1129.00	0.86	0.59	0.01	6.41	0.69	3.89	0.29
353.00	6.03	5.36	1195.00	0.91	0.74	0.03	5.53	0.22	3.23	0.37
353.00	8.02	6.88	1249.00	0.93	0.92	0.04	4.68	0.16	2.65	0.18

Table S8: Densities (ρ in units of kg/m^3), dynamic viscosities (η in units of m Pa s), self-diffusivities of hydrogen (D_{s,H_2}, in units of 10^{-9}m^2/s) and oxygen (D_{s,H_2}, in units of 10^{-9}m^2/s) at different temperatures (T, in units of K), and concentrations of NaOH at 1 bar. The molalities (m, in units of mol/kg) and molarities (C, in units of mol/L) are shown. The standard deviations (σ) are listed for each respective quantity (in the same unit). The TIP4P/2005 water model,[4] the Vrabec H_2 model,[2] the Bohn O_2 model,[5] the Madrid-Transport model[19] (for Na^+), and the newly proposed OH^- model (see the main text) are used for producing these results.

T	m	C	ρ	σ_ρ	η	σ_η	D_{s,H_2}	$\sigma_{D_{s,H_2}}$	D_{s,O_2}	$\sigma_{D_{s,O_2}}$
298.00	1.98	1.98	1081.00	0.92	1.33	0.06	3.05	0.17	1.64	0.12
298.00	3.97	3.94	1157.00	1.56	2.15	0.07	2.10	0.23	1.00	0.08
298.00	6.03	5.94	1228.00	1.33	3.80	0.14	1.31	0.11	0.67	0.08
298.00	8.02	7.80	1291.00	2.15	7.69	0.46	0.74	0.05	0.37	0.04
323.00	1.98	1.95	1069.00	1.73	0.80	0.01	4.66	0.39	2.75	0.25
323.00	3.97	3.90	1143.00	1.29	1.25	0.02	3.75	0.92	1.94	0.22
323.00	6.03	5.86	1212.00	1.07	2.19	0.12	2.41	0.51	1.29	0.08
323.00	8.02	7.71	1275.00	1.28	3.55	0.20	1.47	0.38	0.82	0.05
333.00	1.98	1.94	1063.00	1.12	0.68	0.03	5.89	0.38	3.21	0.52
333.00	3.97	3.87	1136.00	0.59	1.07	0.05	3.99	0.55	2.25	0.19
333.00	6.03	5.83	1206.00	0.79	1.67	0.06	2.85	0.15	1.58	0.08
333.00	8.02	7.66	1267.00	1.15	2.94	0.09	1.85	0.18	1.01	0.06
343.00	1.98	1.93	1056.00	0.99	0.60	0.02	6.89	0.41	4.02	0.15
343.00	3.97	3.85	1129.00	1.32	0.90	0.03	5.05	0.32	2.84	0.28
343.00	6.03	5.79	1198.00	1.38	1.43	0.10	3.17	0.51	1.94	0.13
343.00	8.02	7.61	1260.00	1.95	2.21	0.23	2.14	0.40	1.22	0.13
353.00	1.98	1.92	1050.00	1.29	0.52	0.01	8.07	0.33	4.20	0.47
353.00	3.97	3.82	1122.00	0.20	0.78	0.02	5.61	0.23	3.49	0.21
353.00	6.03	5.76	1191.00	1.22	1.18	0.07	4.12	0.25	2.28	0.12
353.00	8.02	7.57	1253.00	0.86	1.93	0.10	2.63	0.38	1.59	0.17

Table S9: Densities (ρ in units of kg/m^3), dynamic viscosities (η in units of m Pa s), self-diffusivities of hydrogen (D_{s,H_2}, in units of $10^{-9}m^2/s$) and oxygen (D_{s,H_2}, in units of $10^{-9}m^2/s$) at different temperatures (T, in units of K), and concentrations of NaOH at 100 bar. The molalities (m, in units of mol/kg) and molarities (C, in units of mol/L) are shown. The standard deviations (σ) are listed for each respective quantity (in the same unit). The TIP4P/2005 water model,[4] the Vrabec H_2 model,[2] the Bohn O_2 model,[5] the Madrid-Transport model[19] (for Na^+), and the newly proposed OH^- model (see the main text) are used for producing these results.

T	m	C	ρ	σ_ρ	η	σ_η	D_{s,H_2}	$\sigma_{D_{s,H_2}}$	D_{s,O_2}	$\sigma_{D_{s,O_2}}$
298.00	1.98	1.98	1085.00	0.88	1.29	0.04	2.87	0.16	1.52	0.18
298.00	3.97	3.96	1161.00	0.99	2.11	0.12	3.02	0.27	1.04	0.08
298.00	6.03	5.96	1231.00	1.86	3.85	0.21	1.67	0.34	0.69	0.09
298.00	8.02	7.82	1295.00	0.78	7.55	0.14	1.26	0.06	0.40	0.01
323.00	1.98	1.96	1073.00	1.37	0.81	0.04	0.63	0.25	2.74	0.11
323.00	3.97	3.90	1146.00	1.24	1.25	0.04	5.01	0.22	1.95	0.12
323.00	6.03	5.88	1215.00	1.95	2.15	0.08	3.46	0.20	1.23	0.11
323.00	8.02	7.72	1277.00	1.33	3.70	0.31	2.25	0.42	0.80	0.10
333.00	1.98	1.95	1066.00	0.82	0.70	0.03	1.46	0.41	3.25	0.11
333.00	3.97	3.88	1139.00	0.72	1.06	0.09	5.68	0.32	2.36	0.15
333.00	6.03	5.85	1209.00	0.61	1.76	0.07	4.13	0.21	1.53	0.15
333.00	8.02	7.68	1271.00	1.01	2.78	0.11	2.70	0.12	1.03	0.08
343.00	1.98	1.94	1060.00	0.46	0.61	0.02	2.00	0.11	3.99	0.18
343.00	3.97	3.86	1133.00	0.57	0.90	0.04	6.62	0.50	2.69	0.14
343.00	6.03	5.81	1202.00	1.37	1.43	0.05	5.01	0.40	1.99	0.06
343.00	8.02	7.63	1262.00	1.31	2.25	0.04	2.79	1.08	1.19	0.12
353.00	1.98	1.93	1054.00	1.08	0.53	0.02	2.20	0.02	4.37	0.26
353.00	3.97	3.84	1126.00	1.09	0.78	0.02	7.94	0.24	3.37	0.27
353.00	6.03	5.78	1195.00	0.73	1.24	0.04	5.77	0.49	2.31	0.09
353.00	8.02	7.59	1256.00	1.70	1.95	0.03	4.00	0.16	1.59	0.09

Table S10: Excess chemical potentials (μ_{ex} in units of k_BT), Henry coefficients (H in units of 10^3 bar), solubilities (S, in units of 10^{-3}mol/L) of hydrogen (H_2) and oxygen (O_2) at different temperatures (T, in units of K), and concentrations of KOH at 1 bar. The molalities (m, in units of mol/kg) and molarities (C, in units of mol/L) are listed. The standard deviations (σ) are listed for each respective quantity (in the same unit). The TIP4P/2005 water model,[4] the Marx H_2 model,[1] the Bohn O_2 model,[5] the Madrid-Transport model[19] (for K^+), and the newly proposed OH^- model (see the main text) are used for producing these results.

T	m	C	$\mu_{\mathrm{ex,H_2}}$	$\sigma_{\mu_{\mathrm{ex,H_2}}}$	$H_{\mathrm{H_2}}$	$\sigma_{H_{\mathrm{H_2}}}$	$S_{\mathrm{H_2}}$	$\sigma_{S_{\mathrm{H_2}}}$	$\mu_{\mathrm{ex,O_2}}$	$\sigma_{\mu_{\mathrm{ex,O_2}}}$	$H_{\mathrm{O_2}}$	$\sigma_{H_{\mathrm{O_2}}}$	$S_{\mathrm{O_2}}$	$\sigma_{S_{\mathrm{O_2}}}$
298.00	0.00	0.00	4.06	0.02	1.44	0.03	0.697	0.014	3.546	0.046	0.86	0.039	1.165	0.054
298.00	2.04	1.99	4.58	0.03	2.42	0.08	0.414	0.014	4.2	0.097	1.661	0.16	0.608	0.059
298.00	4.07	3.87	5.08	0.04	3.97	0.15	0.252	0.010	4.727	0.148	2.83	0.428	0.361	0.052
298.00	5.93	5.46	5.42	0.04	5.60	0.21	0.178	0.007	5.115	0.053	4.133	0.222	0.243	0.013
298.00	7.96	7.09	5.80	0.09	8.23	0.74	0.122	0.011	5.726	0.069	7.617	0.528	0.132	0.009
323.00	0.00	0.00	4.05	0.02	1.54	0.03	0.651	0.012	3.721	0.038	1.11	0.043	0.902	0.034
323.00	2.04	1.97	4.53	0.02	2.50	0.04	0.401	0.007	4.308	0.03	1.995	0.061	0.502	0.015
323.00	4.07	3.82	4.89	0.03	3.56	0.12	0.281	0.010	4.78	0.092	3.212	0.305	0.314	0.028
323.00	5.93	5.39	5.18	0.02	4.78	0.10	0.209	0.005	5.291	0.073	5.345	0.401	0.188	0.013
323.00	7.96	6.99	5.56	0.03	6.96	0.23	0.144	0.005	5.623	0.072	7.449	0.543	0.135	0.010
333.00	0.00	0.00	4.02	0.02	1.54	0.02	0.648	0.010	3.725	0.049	1.149	0.058	0.872	0.042
333.00	2.04	1.95	4.44	0.03	2.34	0.06	0.428	0.011	4.332	0.057	2.11	0.121	0.475	0.027
333.00	4.07	3.79	4.82	0.02	3.45	0.08	0.290	0.007	4.76	0.058	3.237	0.195	0.310	0.018
333.00	5.93	5.35	5.17	0.04	4.87	0.17	0.206	0.007	5.229	0.035	5.168	0.181	0.194	0.007
333.00	7.96	6.95	5.46	0.06	6.54	0.38	0.153	0.009	5.572	0.085	7.308	0.628	0.138	0.012
343.00	0.00	0.00	3.99	0.01	1.55	0.02	0.646	0.009	3.778	0.041	1.248	0.052	0.803	0.032
343.00	2.04	1.94	4.43	0.04	2.39	0.09	0.419	0.016	4.309	0.044	2.122	0.092	0.472	0.021
343.00	4.07	3.77	4.79	0.02	3.43	0.07	0.292	0.006	4.782	0.046	3.405	0.154	0.294	0.014
343.00	5.93	5.32	5.05	0.02	4.46	0.08	0.224	0.004	5.169	0.088	5.033	0.457	0.200	0.017
343.00	7.96	6.91	5.35	0.04	6.01	0.22	0.166	0.006	5.578	0.114	7.592	0.875	0.133	0.015
353.00	0.00	0.00	3.95	0.02	1.52	0.03	0.660	0.014	3.765	0.028	1.268	0.035	0.790	0.022
353.00	2.04	1.93	4.35	0.02	2.28	0.04	0.438	0.008	4.246	0.019	2.05	0.039	0.488	0.009
353.00	4.07	3.75	4.69	0.03	3.19	0.08	0.313	0.008	4.745	0.075	3.384	0.255	0.297	0.022
353.00	5.93	5.29	4.99	0.04	4.30	0.18	0.233	0.010	5.085	0.068	4.752	0.328	0.211	0.014
353.00	7.96	6.87	5.26	0.05	5.64	0.29	0.177	0.009	5.497	0.089	7.187	0.643	0.140	0.012

Table S11: Excess chemical potentials (μ_{ex} in units of k_BT), Henry coefficients (H in units of 10^3 bar), solubilities (S, in units of 10^{-3}mol/L) of hydrogen (H_2) and oxygen (O_2) at different temperatures (T, in units of K), and concentrations of NaOH at 1 bar. The molalities (m, in units of mol/kg) and molarities (C, in units of mol/L) are listed. The standard deviations (σ) are listed for each respective quantity (in the same unit). The TIP4P/2005 water model,[4] the Marx H_2 model,[1] the Bohn O_2 model,[5] the Madrid-Transport model[19] (for Na^+), and the newly proposed OH^- model (see the main text) are used for producing these results.

T	m	C	μ_{ex,H_2}	$\sigma_{\mu_{\text{ex},H_2}}$	H_{H_2}	$\sigma_{H_{H_2}}$	S_{H_2}	$\sigma_{S_{H_2}}$	μ_{ex,O_2}	$\sigma_{\mu_{\text{ex},O_2}}$	H_{O_2}	$\sigma_{H_{O_2}}$	S_{O_2}	$\sigma_{S_{O_2}}$
298.00	0.00	0.00	4.06	0.02	1.44	0.03	0.697	0.014	3.546	0.046	0.86	0.039	1.165	0.054
298.00	2.04	2.04	4.63	0.03	2.54	0.09	0.394	0.013	4.234	0.117	1.722	0.204	0.589	0.068
298.00	4.07	4.07	5.21	0.08	4.56	0.35	0.221	0.017	4.744	0.15	2.878	0.44	0.355	0.052
298.00	5.93	5.87	5.56	0.09	6.44	0.61	0.157	0.014	5.218	0.214	4.687	1.101	0.223	0.043
298.00	7.96	7.79	6.04	0.12	10.50	1.28	0.097	0.011	5.884	0.322	9.348	2.777	0.119	0.040
323.00	0.00	0.00	4.05	0.02	1.54	0.03	0.652	0.012	3.721	0.038	1.11	0.043	0.902	0.034
323.00	2.04	2.01	4.54	0.03	2.51	0.07	0.399	0.012	4.355	0.071	2.097	0.152	0.479	0.034
323.00	4.07	4.01	5.02	0.06	4.09	0.23	0.246	0.015	4.829	0.083	3.37	0.278	0.299	0.025
323.00	5.93	5.79	5.47	0.06	6.40	0.38	0.157	0.009	5.379	0.12	5.862	0.67	0.173	0.022
323.00	7.96	7.68	5.89	0.10	9.72	1.00	0.104	0.010	5.735	0.122	8.376	1.039	0.121	0.014
333.00	0.00	0.00	4.02	0.02	1.54	0.02	0.648	0.010	3.725	0.049	1.149	0.058	0.872	0.042
333.00	2.04	2.00	4.48	0.05	2.45	0.13	0.409	0.021	4.365	0.041	2.179	0.088	0.460	0.019
333.00	4.07	3.99	4.95	0.03	3.92	0.12	0.255	0.008	4.847	0.105	3.544	0.368	0.285	0.030
333.00	5.93	5.76	5.40	0.06	6.13	0.38	0.164	0.010	5.322	0.035	5.676	0.198	0.176	0.006
333.00	7.96	7.64	5.79	0.08	9.06	0.68	0.111	0.009	5.797	0.167	9.254	1.63	0.111	0.017
343.00	0.00	0.00	3.99	0.01	1.55	0.02	0.647	0.009	3.778	0.041	1.248	0.052	0.803	0.032
343.00	2.04	1.99	4.43	0.03	2.38	0.08	0.420	0.013	4.282	0.037	2.066	0.076	0.485	0.018
343.00	4.07	3.97	4.89	0.01	3.81	0.03	0.263	0.002	4.83	0.044	3.575	0.16	0.280	0.012
343.00	5.93	5.72	5.30	0.05	5.70	0.29	0.176	0.009	5.306	0.059	5.757	0.33	0.174	0.010
343.00	7.96	7.60	5.68	0.04	8.39	0.33	0.119	0.005	5.861	0.103	10.06	1.025	0.101	0.010
353.00	0.00	0.00	3.95	0.02	1.52	0.03	0.660	0.014	3.765	0.028	1.268	0.035	0.790	0.022
353.00	2.04	1.98	4.40	0.02	2.39	0.05	0.419	0.009	4.28	0.015	2.12	0.033	0.472	0.007
353.00	4.07	3.94	4.81	0.03	3.61	0.10	0.277	0.008	4.813	0.068	3.62	0.24	0.278	0.019
353.00	5.93	5.69	5.19	0.01	5.28	0.06	0.190	0.002	5.315	0.139	6.027	0.849	0.169	0.023
353.00	7.96	7.56	5.62	0.06	8.07	0.45	0.124	0.007	5.802	0.105	9.764	1.033	0.104	0.011

Literature Cited

(1) Marx, D.; Nielaba, P. Path-integral Monte Carlo techniques for rotational motion in two dimensions: Quenched, annealed, and no-spin quantum-statistical averages. *Physical Review A* **1992**, *45*, 8968.

(2) Koster, A.; Thol, M.; Vrabec, J. Molecular models for the hydrogen age: hydrogen, nitrogen, oxygen, argon, and water. *Journal of Chemical & Engineering Data* **2018**, *63*, 305–320.

(3) Celebi, A. T.; Jamali, S. H.; Bardow, A.; Vlugt, T. J. H.; Moultos, O. A. Finite-size effects of diffusion coefficients computed from molecular dynamics: a review of what we have learned so far. *Molecular Simulation* **2021**, *47*, 831–845.

(4) Abascal, J. L.; Vega, C. A general purpose model for the condensed phases of water: TIP4P/2005. *The Journal of Chemical Physics* **2005**, *123*, 234505.

(5) Bohn, M.; Lustig, R.; Fischer, J. Description of polyatomic real substances by two-center Lennard-Jones model fluids. *Fluid Phase Equilibria* **1986**, *25*, 251–262.

(6) Zeron, I.; Abascal, J.; Vega, C. A force field of Li^+, Na^+, K^+, Mg^{2+}, Ca^{2+}, Cl^-, and SO_4^{2-} in aqueous solution based on the TIP4P/2005 water model and scaled charges for the ions. *The Journal of Chemical Physics* **2019**, *151*, 134504.

(7) Rahbari, A.; Hens, R.; Ramdin, M.; Moultos, O. A.; Dubbeldam, D.; Vlugt, T. J. H. Recent advances in the Continuous Fractional Component Monte Carlo methodology. *Molecular Simulation* **2021**, *47*, 804–823.

(8) Shi, W.; Maginn, E. J. Continuous Fractional Component Monte Carlo: an adaptive biasing method for open system atomistic simulations. *Journal of Chemical Theory and Computation* **2007**, *3*, 1451–1463.

(9) Shi, W.; Maginn, E. J. Improvement in molecule exchange efficiency in Gibbs ensemble Monte Carlo: Development and implementation of the Continuous Fractional Component move. *Journal of Computational Chemistry* **2008**, *29*, 2520–2530.

(10) Hens, R.; Rahbari, A.; Caro-Ortiz, S.; Dawass, N.; Erdős, M.; Poursaeidesfahani, A.; Salehi, H. S.; Celebi, A. T.; Ramdin, M.; Moultos, O. A.; Dubbeldam, D.; Vlugt, T. J. H. Brick-CFCMC: Open Source Software for Monte Carlo Simulations of Phase and Reaction Equilibria Using the Continuous Fractional Component Method. *Journal of Chemical Information and Modeling* **2020**, *60*, 2678–2682.

(11) Polat, H. M.; Salehi, H. S.; Hens, R.; Wasik, D. O.; Rahbari, A.; de Meyer, F.; Houriez, C.; Coquelet, C.; Calero, S.; Dubbeldam, D.; Moultos, O. A.; Vlugt, T. J. H. New Features of the Open Source Monte Carlo Software Brick-CFCMC: Thermodynamic Integration and Hybrid Trial Moves. *Journal of Chemical Information and Modeling* **2021**, *61*, 3752–3757.

(12) Walker Jr, R. D. *Study of gas solubilities and transport properties in fuel cell electrolytes*; Technical Report, Florida Univ., Gainesville (USA), 1971.

(13) Young, C. L. *Solubility Data Series: Hydrogen and Deuterium*, 1st ed.; Pergamon Press: Oxford, 1981; Vol. 5/6.

(14) Olsson, J.; Jernqvist, Å.; Aly, G. Thermophysical properties of aqueous NaOH-H_2O solutions at high concentrations. *International Journal of Thermophysics* **1997**, *18*, 779–793.

(15) Tsimpanogiannis, I. N.; Moultos, O. A.; Franco, L. F.; Spera, M. B. d. M.; Erdős, M.; Economou, I. G. Self-diffusion coefficient of bulk and confined water: a critical review of classical molecular simulation studies. *Molecular Simulation* **2019**, *45*, 425–453.

(16) Tsimpanogiannis, I. N.; Maity, S.; Celebi, A. T.; Moultos, O. A. Engineering Model for Predicting the Intradiffusion Coefficients of Hydrogen and Oxygen in Vapor, Liquid, and

Supercritical Water based on Molecular Dynamics Simulations. *Journal of Chemical & Engineering Data* **2021**, *66*, 3226–3244.

(17) Ryckaert, J.; Ciccotti, G.; Berendsen, H. J. Numerical integration of the cartesian equations of motion of a system with constraints: molecular dynamics of n-alkanes. *Journal of Computational Physics* **1977**, *23*, 327–341.

(18) Plimpton, S. Fast parallel algorithms for short-range molecular dynamics. *Journal of Computational Physics* **1995**, *117*, 1–19.

(19) Blazquez, S.; Conde, M. M.; Vega, C. in Preparation. **2023**,

(20) Gilliam, R.; Graydon, J.; Kirk, D.; Thorpe, S. A review of specific conductivities of potassium hydroxide solutions for various concentrations and temperatures. *International Journal of Hydrogen Energy* **2007**, *32*, 359–364.

(21) Guo, Y.; Xu, H.; Guo, F.; Zheng, S.; Zhang, Y. Density and viscosity of aqueous solution of K_2CrO_4/KOH mixed electrolytes. *Transactions of Nonferrous Metals Society of China* **2010**, *20*, s32–s36.

(22) Yeh, I.; Hummer, G. System-size dependence of diffusion coefficients and viscosities from molecular dynamics simulations with periodic boundary conditions. *The Journal of Physical Chemistry B* **2004**, *108*, 15873–15879.

(23) Dünweg, B.; Kremer, K. Molecular dynamics simulation of a polymer chain in solution. *The Journal of Chemical Physics* **1993**, *99*, 6983–6997.

(24) Jamali, S. H.; Bardow, A.; Vlugt, T. J. H.; Moultos, O. A. Generalized form for finite-size corrections in mutual diffusion coefficients of multicomponent mixtures obtained from equilibrium molecular dynamics simulation. *Journal of Chemical Theory and Computation* **2020**, *16*, 3799–3806.

(25) Tham, M. J.; Walker Jr, R. D.; Gubbins, K. E. Diffusion of oxygen and hydrogen in aqueous potassium hydroxide solutions. *The Journal of Physical Chemistry* **1970**, *74*, 1747–1751.
